# Evolution of the Quinoline Scaffold for the Treatment of Leishmaniasis: A Structural Perspective

**DOI:** 10.3390/ph17030285

**Published:** 2024-02-22

**Authors:** Carlos F. M. Silva, Diana C. G. A. Pinto, Pedro A. Fernandes, Artur M. S. Silva

**Affiliations:** 1 LAQV-REQUIMTE, Department of Chemistry, University of Aveiro, 3810-193 Aveiro, Portugal; silva.c@ua.pt (C.F.M.S.); artur.silva@ua.pt (A.M.S.S.); 2 UCIBIO, REQUIMTE, Departamento de Química e Bioquímica, Faculdade de Ciências, Universidade do Porto, 4169-007 Porto, Portugal; pafernan@fc.up.pt

**Keywords:** leishmania, *N*-heterocycles, quinolines, promastigotes, amastigotes

## Abstract

Since the beginning of the XXI century, Leishmaniasis has been integrated into the World Health Organization’s list of the 20 neglected tropical diseases, being considered a public health issue in more than 88 countries, especially in the tropics, subtropics, and the Mediterranean area. Statistically, this disease presents a world prevalence of 12 million cases worldwide, with this number being expected to increase shortly due to the 350 million people considered at risk and the 2–2.5 million new cases appearing every year. The lack of an appropriate and effective treatment against this disease has intensified the interest of many research groups to pursue the discovery and development of novel treatments in close collaboration with the WHO, which hopes to eradicate it shortly. This paper intends to highlight the quinoline scaffold’s potential for developing novel antileishmanial agents and provide a set of structural guidelines to help the research groups in the medicinal chemistry field perform more direct drug discovery and development programs. Thus, this review paper presents a thorough compilation of the most recent advances in the development of new quinoline-based antileishmanial agents, with a particular focus on structure–activity relationship studies that should be considerably useful for the future of the field.

## 1. Introduction

By the beginning of the XXI century, the World Health Organization (WHO) published a list of 20 diseases recognized as neglected tropical diseases [1,2]. This novel nomenclature emerged to describe a distinct group of diseases that disproportionately affect populations with poor living conditions, promoting high morbidity and mortality rates. Furthermore, in some cases, these diseases also instigate stigma and discrimination among populations. From these 20 diseases, it is essential to highlight leishmaniasis as one of the most neglected diseases, mainly affecting people from developing countries from the tropics, subtropics, and the Mediterranean basin, with approximately 350 million individuals at risk of developing the disease [3]. This disease constitutes a public health issue in more than 88 countries, presenting an estimated world prevalence of 12 million cases, with 700,000–1,000,000 new cases each year [4].

**Figure 1:**
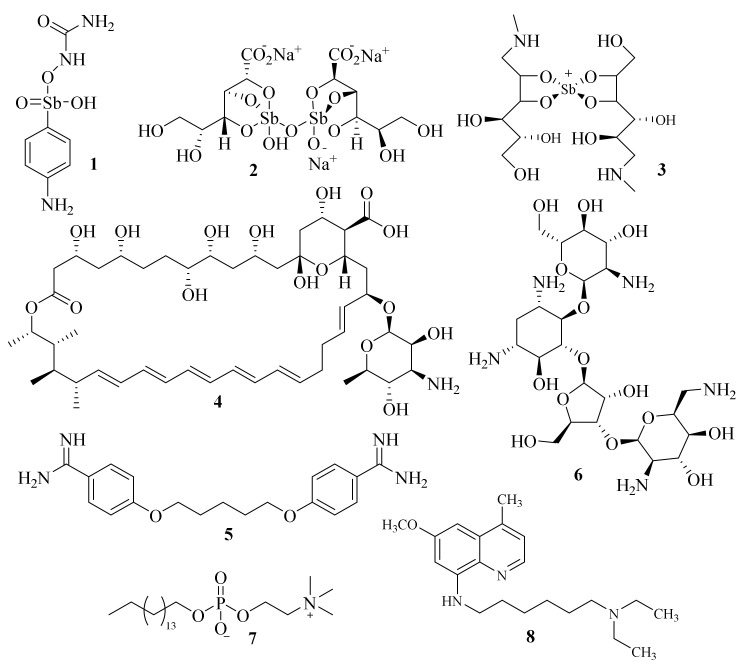
Currently used antileishmanial agents.

Since there is no effective vaccine, the treatment of leishmaniasis is solely dependent on chemotherapy, with the organoantimonial compounds remaining as the first line of treatment for all forms of leishmaniasis [5]. The first effective drug to treat leishmaniasis was urea stibamine (**1**, Figure 1), which was discovered in 1912 but was only described as effective against L. donovani in 1922 [6]. This massive breakthrough would then lead to the development and refinement of pentavalent antimonials [Sb(V) compounds], such as the generic sodium stibogluconate (**2**), also known as pentostam, or the branded meglumine antimoniate (**3**), also known as glucantim, progressively reducing the side effects of the treatments against leishmaniasis. The need for safer and more effective medicines to treat leishmaniasis drove the search for new compounds. Amphotericin B (**4**, AmB, Figure 1) emerged as the first alternative to the usual pentavalent antimonials [3,7]. Furthermore, other antileishmanial agents, such as pentamidine (**5**) [8], paromomycin (**6**) [9], miltefosine (**7**) [10] or sitamaquine (**8**) [11] have also emerged as pharmaceutical alternatives for the treatment of leishmaniasis. However, the use of these antileishmanial drugs has faced several limitations throughout the years, such as the emergence of several side effects or the increased incidence of resistance mechanisms of the parasites [12].

Based on the absence of appropriate treatment and increased resistance against the currently used medicines, the search for novel antileishmanial drugs is starting to be in the spotlight in the scientific community. Throughout the years, quinoline scaffold has been used as the core structure for developing promising antileishmanial agents. There is a long way to go until the discovery of an effective treatment against this disease [13,14,15]. Thus, this paper intends to provide a structural perspective of the use of the quinoline scaffold in the development of novel antileishmanial agents, emphasizing not only the structural modifications crucial for increasing the antileishmanial properties but also the functionalizations performed to improve their pharmacological properties.

## 2. Quinolines as Antileishmanial Agents

**Figure 2:**
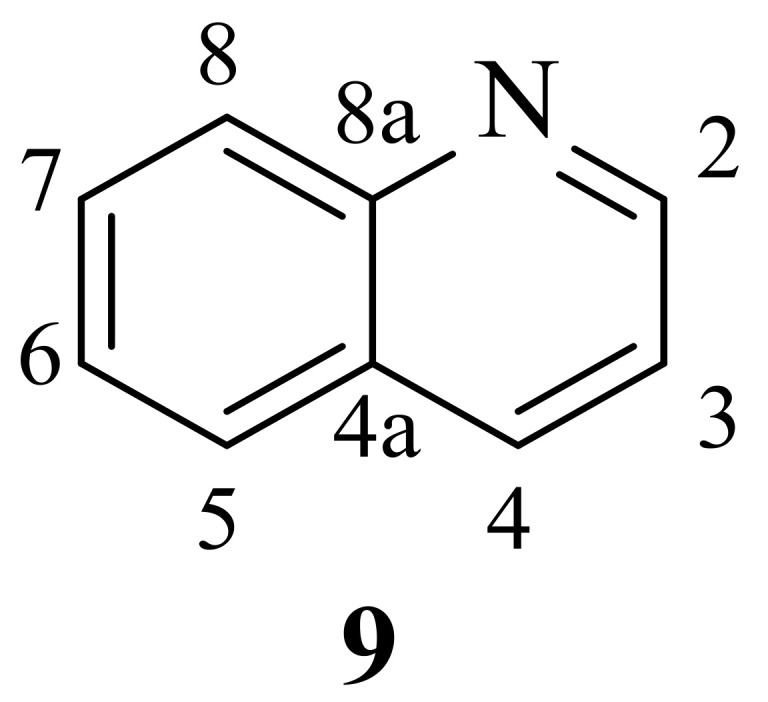
Quinoline scaffold (**9**) and respective numbering system.

Firstly, discovered by F. F. Runge, in 1834, quinoline (**9**) appeared as a colorless hygroscopic liquid isolated by the distillation of coal tar, its structure only being revealed by Dewar, in 1871 [16,17]. Dewar observed the chemical similarity between pyridine and quinoline and described quinoline’s structure as a rigid heterocyclic core of benzene *ortho*-fused with a pyridine ring (Figure 2).

**Figure 3:**
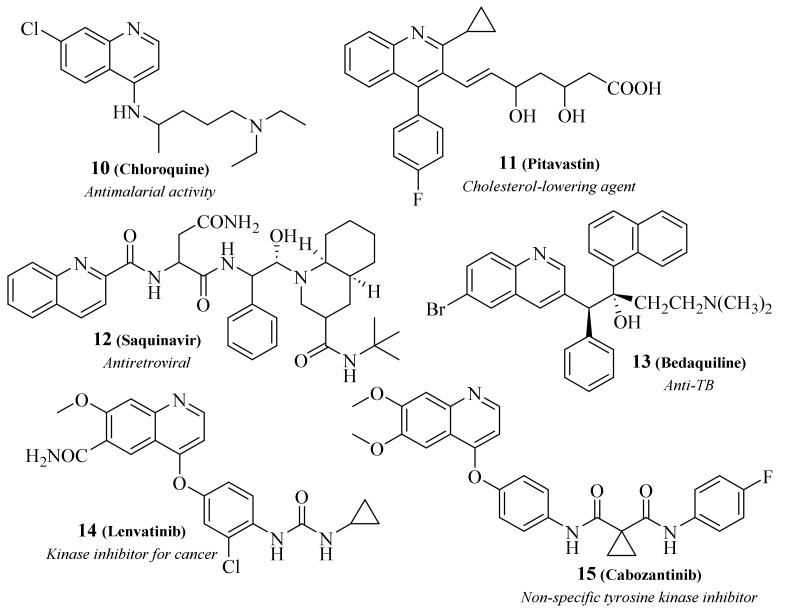
Some examples of quinoline-based approved drugs.

Since its discovery, quinoline derivatives, known as 1-azanaphthalene or benzo[*b*]pyridine, have been associated with various pharmacological applications [18,19]. Considering this fact, extensive libraries of quinoline derivatives have been reported for their significant biological properties, such as antibacterial, antifungal, antiviral, antiprotozoal, antimalarial, anti-inflammatory, and anticancer, amongst others [19]. It is important to mention that these properties have led the quinoline scaffold to be the main structural feature of many approved drugs since the discovery of the widely used antimalarial chloroquine in 1934 by H. Andersag et al. at the Bayer laboratories (**10**, Figure 3). Some of the other quinoline-based approved drugs include pitavastin (**11**), saquinavir (**12**), bedaquiline (**13**), lenvatinib (**14**), cabozantinib (**15**) and many others.

In addition to the aforementioned activities, quinolines have also been found to possess promising antileishmanial activity, with sitamaquine (**8**, Figure 1) being developed as a promising medicine for the oral treatment of leishmaniasis [11]. However, due to the side effects shown during the phase 2-b randomized clinical trials, its further development was terminated in 2017. Since the discovery of sitamaquine (**8**), numerous examples of quinolines have been described as having promising antileishmanial activities, with some review articles already depicting some of these promising molecules [19,20,21,22]. However, this paper intends to provide a different and more complete insight into the real potential of quinoline derivatives for developing novel antileishmanial treatments. Our approach will mostly focus on compiling all the structural information already described to facilitate access to this type of knowledge in the medicinal chemistry without disregarding the information gathered in previous review articles.

### 2.1. Quinoline Derivatives as Antileishmanial Agents until Mid-2013 [20]

Until 2013, numerous quinolines have been evaluated for their potential for developing novel antileishmanial treatments, leading to the compilation of this knowledge in a complete review published by Reynolds et al. [20]. In this review article, the authors identified the most promising quinoline-based antileishmanial agents already reported and gathered the structural information into a structure-activity relationship (SAR) study. This compilation included the analysis of mono-, di- and tri- and poly-substituted (tetra- and penta-) quinolines, presenting for each of these groups the main structural features associated with the compounds’ antileishmanial properties. 

**Figure 4:**
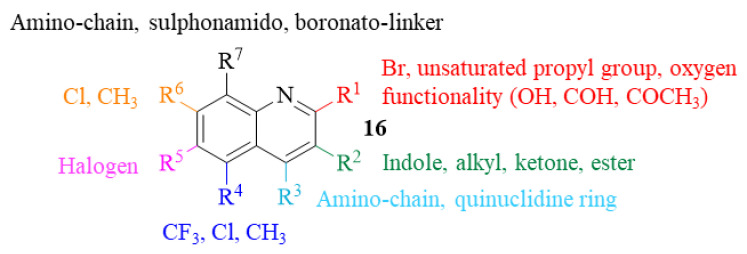
Structural–antileishmanial activity relationship study of quinoline derivatives (**16**) reported until mid-2013 [20].

Like what we faced while preparing this paper, Reynolds et al. emphasize the main drawbacks of analyzing and comparing results from different studies. These difficulties include the fact that different stages of the parasite (promastigote and amastigote) are indiscriminately used to evaluate the compounds’ antileishmanial activity in vitro, as well as distinct in vivo models (mouse, canine, rat or hamster). Furthermore, the units used to quantify the antileishmanial activity vary from study to study, considerably interfering with a proper comparison between compounds. The absence of a clear understanding of the compound’s mechanism of action or lack of identification of the molecular target also impairs the comparison. Different derivatives can act against the same target, and the same derivative might act against numerous different targets of pathways. As a qualitative guide, these authors presented a SAR study trying to go further than the obvious remark that substitutions at any position of the quinoline scaffold can potentially originate derivatives with improved antileishmanial properties (**16**, Figure 4). In addition, the description of the leading quinoline-based derivatives being developed at that time and undergoing preclinical and clinical studies is also made, allowing the medicinal chemistry field to fully understand the stance of the antileishmanial drug discovery and development pipeline.

### 2.2. Quinoline Derivatives as Antileishmanial Agents Post-2013

Despite the huge amount of information available by mid-2013, the interest of many medicinal chemistry research groups in developing promising novel antileishmanial quinoline derivatives was still accentuated. This interest led numerous research groups to continue making significant contributions to developing antileishmanial treatments based on quinoline derivatives, as this subtopic will thoroughly depict.

**Figure 5:**
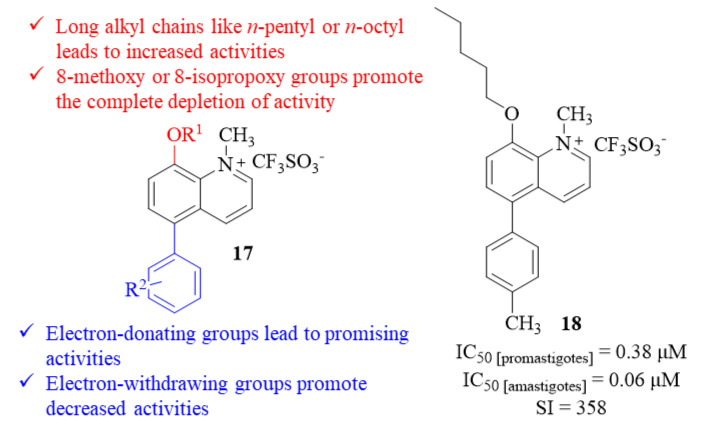
Structure-antileishmanial activity relationship of quinolinium salts (**17**), with an emphasis on derivative **18**.

In 2013, while developing a novel rapid drug screening assay for antileishmanial activity, Bringmann et al. synthesized and evaluated a series of forty-nine quinolinium salts against both *L. major* promastigotes and amastigotes [23]. Considering their effect on promastigotes, the results demonstrated that only twenty quinolinium salts from the entire series of evaluated compounds have considerable antileishmanial activities (IC_50_ < 10 µM), with the remaining derivatives showing low to no activities. Based on the results, a SAR study was also delineated to understand the most critical structural moieties for the quinolinium salts’ antileishmanial properties. Introducing an 8-methoxy or an 8-isopropoxy function promotes the complete depletion of activity, while longer alkyl chains like pentyl or octyl clearly increase the compounds’ biological activity. The influence of the 5-substitutions was also considerably evaluated, particularly the effect of the functional group in a 5-aryl fragment. This evaluation demonstrated that an aryl fragment, containing an electron-donating substituent, promotes promising antileishmanial activities, while an aryl fragment with an electron-withdrawing substituent promotes low activity levels (**17**, Figure 5). Only seven of these twenty promising compounds were further evaluated against *L. major* amastigotes infecting blood marrow-derived macrophages (BDMD). This final evaluation showed that six displayed high activity levels against intracellular amastigotes (IC_50_ < 0.3 µM). Despite not being the most active compound against amastigotes, the potential of derivative **18** must be emphasized since it presents an IC_50_ of 0.06 µM and a selectivity index of 358 (Figure 5). 

**Figure 6:**
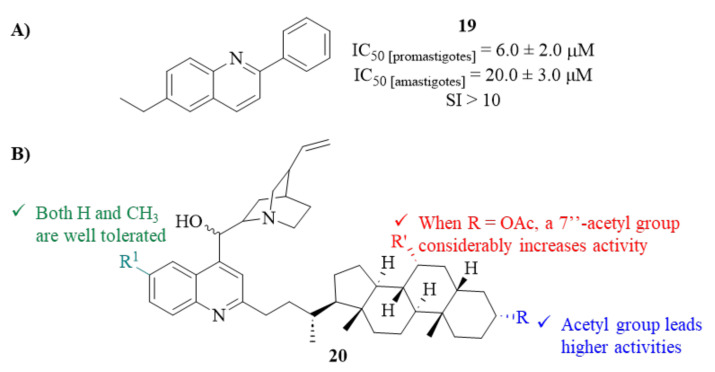
(**A**) Most promising 2-arylquinoline derivative (**19**) against *L. amazonensis*; (**B**) Structure-antileishmanial activity relationship study of *Cinchona* alkaloid-bile acid hybrids (**20**) against *L. mexicana* promastigotes.

In the same year, through a screening program for new biologically active *N*-heterocyclic compounds, Bompart et al. synthesized and evaluated a series of 2-aryl quinolines as promising antileishmanial agents against *L. braziliensis* promastigotes and amastigotes [24]. From this series of seven derivatives, only one compound (**19**, Figure 6) demonstrates, simultaneously, considerable effects against promastigotes (IC_50_ = 6.0 ± 2.0 µM) and amastigotes (IC_50_ = 20 ± 2.0 µM), in addition to low levels of toxicity against BMDM macrophages (IC_50_ > 200 µM). Following the promising profile of this derivative, its mechanism of action was also evaluated, particularly the evaluation of its effects on the parasite bioenergetics and sterol biosynthetic pathway. Considering its influence on the parasite bioenergetics, the results led this research group to suggest that this derivative’s cationic nature might activate an electrophoretic mechanism that compromises the parasite’s viability by promoting the failure of the mitochondrial potential. In turn, regarding the sterol biosynthetic pathway, this work was able to demonstrate an accumulation of squalene and a depletion of 5-dehydroepisterol in treated parasites, an effect that had already been described for miltefosine against *L. mexicana* [25].

Based on the principle of bioconjugation/hybridization, which allows the formation of hybrid compounds with the combined properties of their individual components by binding two or more active molecules, another research group prepared a series of sixteen new *Cinchona* alkaloid-bile acid hybrids and evaluated them against *L. mexicana* promastigotes (**20**, Figure 6) [26]. The results demonstrated that, essentially, the entire compound series demonstrated considerable antileishmanial activity values (IC_50_ < 20 µM). Interestingly, considering the group of acetylated molecules, one was able to verify that the presence of a 7″-acetyl group considerably increases the antileishmanial activity of these compounds (IC_50_ from 18.08–20.04 to 4.62–5.50 µM). Considering the group of deacetylated derivatives, most of the derivatives showed the same range of activity levels (IC_50_ = 5.29–6.96 µM), being even more active than the correspondent *cinchona* alkaloid precursors. However, this series of molecules also revealed high levels of toxicity against normal human fibroblast cell lines (WI-38), originating SI > 1, which makes this type of compound unsuitable for use in further developments of antileishmanial drugs.

Following a previous study by Alain Fournet et al. [27], where several structurally simple 2-substituted quinolines were described as having in vitro and in vivo antiparasitic properties, another research group decided to further develop this type of derivative. Using two of the already identified molecules as parent compounds, Gopinath et al. prepared a series of 2-substituted quinolines to address the limitations of this previous work, namely the weak in vitro potency and metabolic instability [28]. Through several C-2 modifications on the quinoline ring, it was possible to verify that the two most active derivatives against *L. donovani* intracellular amastigotes were the ones containing the prop-2-en-diol (IC_50_ = 10.04 ± 1.4 µM) and prop-2-enfluoride (IC_50_ = 6.68 ± 1.1 µM) groups. However, in addition to weak potency, this first group of derivatives also demonstrated low solubility levels and/or metabolic stability.

Then, using the prop-2-en-diol-contaning derivative as a core fragment, a second modification had to be performed to overcome these limitations, which was attempted by introducing chloro, fluoro, and methoxy substitution on the quinoline ring. The results demonstrated that considering C-6 modifications, only the introduction of a chloro atom led to a highly active derivative (IC_50_ = 0.86 ± 0.1 µM; SI = 33.59), while the introduction of both fluoro atom or methoxy group promoted a considerable activity decrease (IC_50_ = 17.9 ± 1.4 µM and IC_50_ = 32.38 ± 2.3 µM). Furthermore, while maintaining the 6-Cl atom, the introduction of a fluoro atom at either C-5 or C-7 led to a considerable loss of antileishmanial activity (IC_50_ = 4.1 ± 1.0 µM and IC_50_ = 14.35 ± 2.1 µM, respectively), with this effect being more accentuated with the introduction of a 7-F atom. Despite this activity loss, combining the 6-chloro and 7-fluoro atoms in the quinoline ring promoted lower cytotoxicity levels and significantly improved metabolic stability.

**Figure 7:**
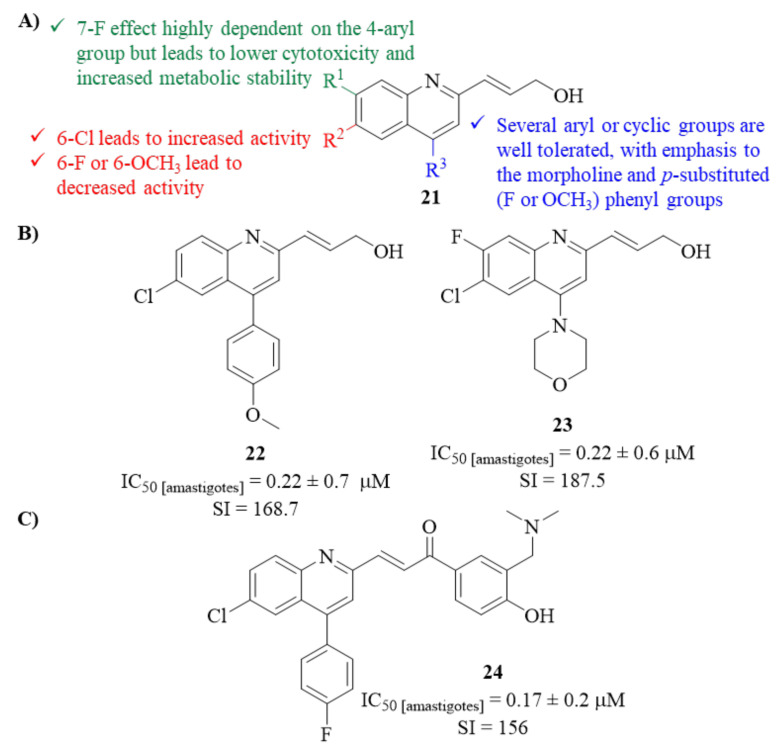
(**A**) Structure-antileishmanial activity relationship study of 2-substituted quinolines (**21**). (**B**) Structures of derivatives **22** and **23**, the two most promising antileishmanial agents. (**C**) Structure of derivative **24**, an antileishmanial agent developed through an optimization process.

Finally, a further optimization process was performed by introducing several 4-aryl groups to assess the relevance of the substitution at this position to the in vitro potency against the parasite. Through this optimization, two derivatives emerged as promising antileishmanial agents, with high levels of activity and low levels of both cytotoxicity and metabolism (**22**, IC_50_ = 0.22 ± 0.7 µM; SI = 168.72 and **23**, IC_50_ = 0.22 ± 0.06 µM; SI = 187.5). Interestingly, during this process, one could verify that the influence of a 7-fluoro atom in the quinoline is highly dependent on the 4-aryl group introduced. For instance, considering derivative **22**, introducing the 7-fluoro atom would considerably decrease the compound’s antileishmanial properties. In contrast, for derivative **23**, the 7-fluoro atom seems crucial for the high levels of activity (Figure 7).

A year later, in 2014, the same research group decided to develop their lead optimization program further by synthesizing a novel series of seven chalcone-type derivatives, with compound **23** as the structural core [29]. This novel series was evaluated for its in vitro antileishmanial activity against *L. donovani* intracellular amastigotes. The results demonstrated that, when compared with miltefosine (IC_50_ = 8.10 ± 0.60 µM; SI = 7), all the evaluated derivatives present higher levels of antileishmanial activity (IC_50_ = 0.17–6.42 µM) and selectivity indexes (SI = 13–156). Furthermore, these new analogues demonstrated similar levels of metabolic stability but lower solubility levels than the precursor (**23**, Figure 6). Structurally, different combinations of the same fragments promoted different levels of antileishmanial activity, impairing the development of a proper SAR study. Nevertheless, considering the substitution on the chalcone fragment, it was possible to verify that the most suitable substituent was the 3′-[(dimethylamino)methyl]-4′-hydroxyphenyl. Finally, the most active compound against *L. donovani* was compound **24** (Figure 7), which possessed the combination of a 4-fluorophenyl in the quinoline ring and a 3′-[(dimethylamino)methyl]-4′-hydroxyphenyl in the chalcone fragment (IC_50_ = 0.17 ± 0.02 µM; SI = 156).

**Figure 8:**
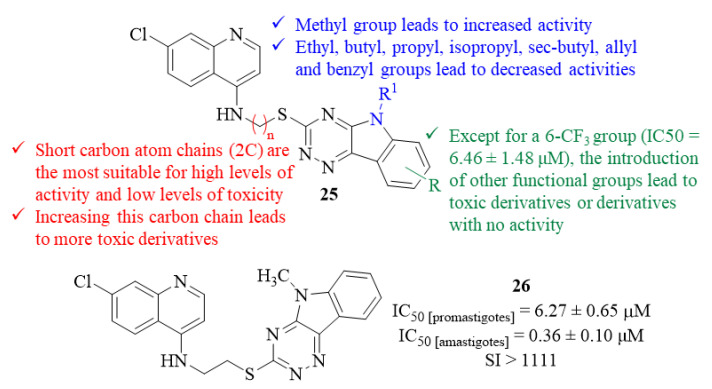
Structure-antileishmanial activity relationship study of triazino indole-quinoline hybrids (**25**), with particular emphasis on derivative **26**.

Still, in 2013, another research group decided to implement a hybridization approach for the development of novel antileishmanial agents against *L. donovani* promastigotes and amastigotes, leading to the synthesis and evaluation of a series of nineteen triazino indole-quinoline hybrids [30]. The antileishmanial evaluation of this series of derivatives, especially against the intracellular stage of the parasite, allowed the development of a SAR study with particular focus on three major structural features: (i) the chain length of the linker between the two pharmacophores; (ii) the *N*-alkyl group introduced in the triazino indole fragment; and (iii) the substitution pattern on the indole’s aromatic ring (C-6 and C-8). Thus, considering the chain length of the linker, one could verify that derivatives with short carbon atom chains (2C) between the two pharmacophores were the most suitable compounds to be used as antileishmanial agents by simultaneously presenting high levels of activity (IC_50_ = 0.36 ± 0.10–7.10 ± 1.27 µM) and considerable selectivity indexes (SI from 7 to >1111). Interestingly, increasing this carbon chain makes this type of derivative toxic to the J-774A.1 macrophage cells while maintaining considerable antileishmanial activity. Furthermore, considering the *N*-alkyl group introduced in the triazino indole fragment, only the introduction of an *N*-methyl group promoted an activity increase from 1.11 ± 0.19 µM to 0.36 ± 0.10 µM. In turn, the remaining functional groups introduced, such as ethyl, butyl, propyl, isopropyl, sec-butyl, allyl and benzyl, originated lower activity levels with IC_50_ values as high as 29.48 ± 2.58 µM. Finally, regarding the substitution pattern on the indole’s aromatic ring (C-6 and C-8), except for the introduction of a 6-CF_3_ group (IC_50_ = 6.46 ± 1.48 µM), the introduction of any other functional group led to toxic derivatives or derivatives with no antileishmanial activity (Figure 8).

**Figure 9:**
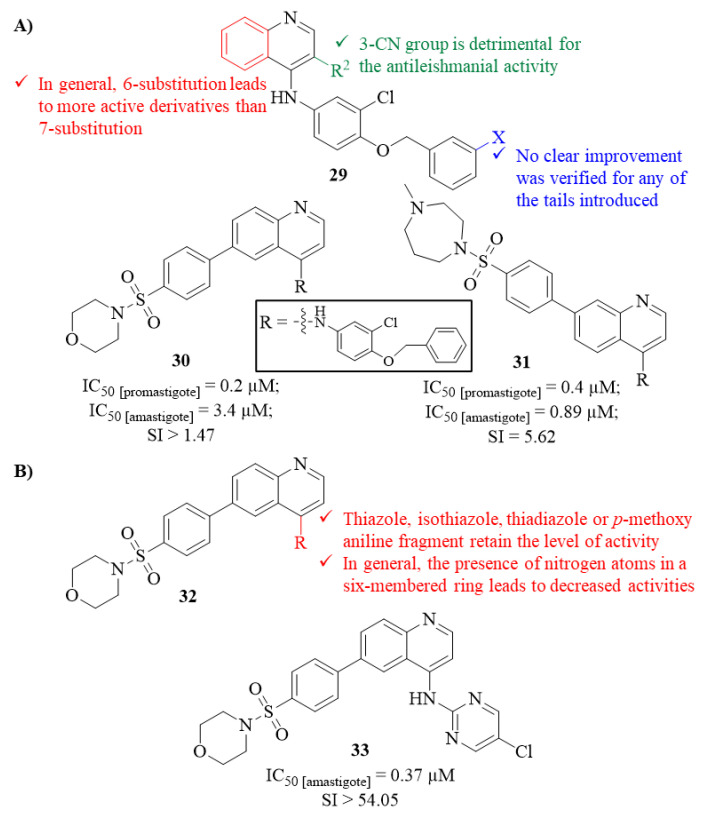
(**A**) Structure-antileishmanial activity relationship study (**29**) with a particular emphasis on derivatives **30** and **31** for being the most active analogs against *L. major* promastigotes and amastigotes, respectively. (**B**) Structure-antileishmanial activity relationship study (**32**) of the optimization process, with particular emphasis on derivative **33**.

Two years later, in 2015, Devine et al. performed a considerable screening assay against several parasites, such as *T. cruzi* (Chagas disease), *L. major* (leishmaniasis), and *P. falciparum* (malaria), evaluating numerous compounds from seven different scaffolds [31]. From this entire library of compounds, and narrowing this work to the antileishmanial evaluation, two groups of quinoline derivatives must be highlighted for their antileishmanial potential against both stages of *L. major* (**29**, Figure 9). The results demonstrated that several quinoline derivatives were able to impair the growth and survival of both promastigotes considerably (IC_50_ 0.2–4.1 µM) and amastigotes (IC_50_ 0.89–4.0 µM), with particular emphasis on the derivatives from the first group. The higher levels of antileishmanial activity observed for most of the derivatives from the first group, in comparison with the corresponding analogues from the second group, suggest that the presence of a 3-CN group is detrimental to the antileishmanial properties of this type of compound. Furthermore, by comparing the quinoline derivatives with the corresponding quinazoline and isoquinoline analogues, it was possible to verify that the sole presence of the N1 appears to be essential for their antipromastigote properties as both quinazoline (IC_50_ = 0.50 µM) and isoquinoline (IC_50_ > 15 µM) analogues present lower levels of activity than the quinoline correspondent (IC_50_ = 0.20 µM). Considering the tail variations performed in these two groups of quinoline series, no clear improvement was verified for any of the tails introduced, suggesting that additional work should be performed to find the optimal core structure to promote higher activity levels. Finally, from this series of sixteen derivatives, analogues **30** (IC_50_ [promastigote] = 0.2 µM and IC_50_ [amastigote] = 3.4 µM) and **31** (IC_50_ [promastigote] = 0.4 µM and IC_50_ [amastigote] = 0.89 µM) should be highlighted as the most potent derivatives against promastigotes and amastigotes, respectively (Figure 9).

Following the identification of derivative **31** as a lead molecule against leishmaniasis in 2017, this group focused on developing new analogues to improve its poor drug-like properties while maintaining or improving in vitro activity against *L. major* intracellular amastigotes [32]. For this optimization work, twenty-seven novel analogues were synthesized and evaluated against *L. major* intracellular amastigotes with different functional groups introduced at C-4 of the quinoline scaffold. The results demonstrated that the analogues containing a thiazole, isothiazole, thiadiazole or *p*-methoxy aniline fragment can maintain the antileishmanial activity in the same micromolar range (1.2−5.8 µM). In turn, apart from two analogues, the presence of nitrogen atoms in a six-membered ring considerably decreases the antileishmanial activity of this type of compound (Figure 9). Interestingly, one of the exceptions emerged as the most active from this series of analogues, being the only compound able to achieve submicromolar antileishmanial activity (**33**, IC_50_ = 0.37 µM, Figure 9).

**Figure 10:**
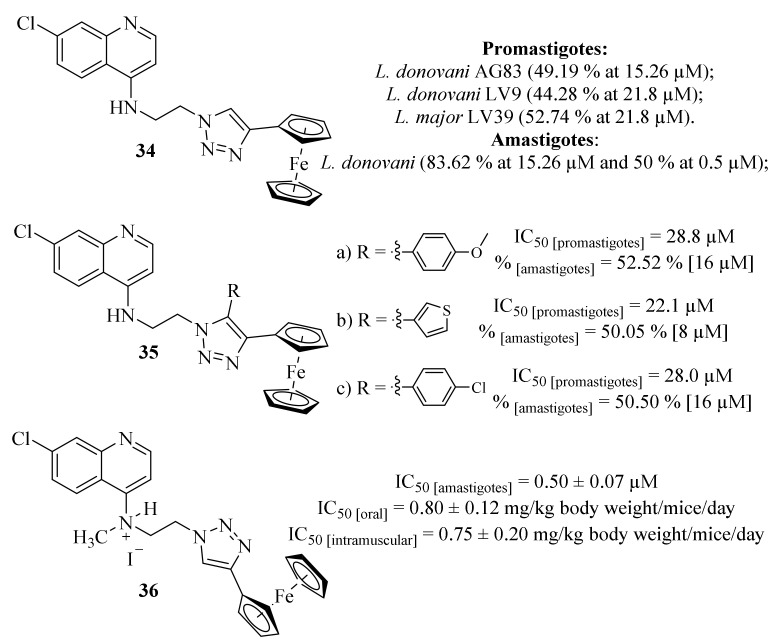
Ferrocenylquinoline derivatives (**34**–**36**), organometallic hybrids with promising antileishmanial properties against *L. donovani* and *L. major*.

Also, in 2015, Yousuf et al. were particularly focused on the development of organometallic quinoline derivatives as novel antileishmanial agents through the synthesis and antileishmanial evaluation of ferrocenylquinoline (**34**) against *L. donovani* and *L. major* (Figure 10) [33]. Considering its effects against *L. donovani* and *L. major* promastigotes, the results demonstrated that ferrocenylquinoline (**34**) was able to inhibit the proliferation of the parasite in a dose-dependent manner, being more effective against *L. donovani* AG83 (49.19% at 15.26 µM) and even more potent than miltefosine (IC_50_ = 21 µM) against this particular species. Furthermore, ferrocenylquinoline (**34**) was also able to inhibit the growth and proliferation of *L. donovani LV9* (44.28%) and *L. major LV39* (52.74%) at 21.8 µM. Regarding the amastigote stage of *L. donovani*, this compound was also able to considerably affect the amastigotes in 50% at 0.5 µM, while also significantly increasing the level of NO in infected macrophages. This research group then identified a compound with promising antileishmanial properties, suggesting that these effects might be associated with its ability to induce parasite death by promoting oxidative stress and depolarizing mitochondrial membrane potential.

Following their work, this research group focused on synthesizing a series of thirteen ferrocenylquinoline derivatives as promising antileishmanial agents against *L. donovani *promastigotes and amastigotes [34]. From this series of thirteen derivatives, three of them presented promising antileishmanial activities against *L. donovani* AG83 promastigotes (IC_50_ [**35.a**] = 28.7 µM, IC_50_ [**35.b**] = 22.1 µM and IC_50_ [**35.c**] = 28.0 µM), with the remaining derivatives presenting IC_50_ values above 32 µM (Figure 10). The higher antileishmanial activity of derivative **35.b** might be related to the presence of the thiophene fragment since this type of nucleus provides conjugation for electron delocalization pathways more efficiently than phenyl groups, which may promote chain reactions and additional redox properties for these compounds. Then, the three most active compounds were selected for further evaluation, including their effects on cell cycle arrest and apoptosis and their effects against intracellular amastigotes and induction of NO. These further evaluations demonstrated that these derivatives appear to induce cell apoptosis on *L. donovani* promastigotes as well as promote the generation of NO, which is considered the primary effector molecule of a pro-inflammatory response leading to the suppression of *L. donovani* amastigote in the infected macrophages. Furthermore, these three derivatives were also demonstrated to be considerably effective against *L. donovani* amastigotes, being able to inhibit its growth and proliferation by 52.51% [16 µM], 50.05% [8 µM] and 50.50% [16 µM], respectively.

A few years later, and following the promising results already described, this research group decided to compile the advantages of both quinoline and ferrocene scaffolds for the development of antileishmanial agents by synthesizing a novel series of four water-soluble ferrocenyl quinoline derivatives (Figure 10) [35]. In this work, the series of ferrocenylquinoline derivatives was evaluated for its effects against *L. donovani* amastigotes, demonstrating that all derivatives present promising antileishmanial activities (IC_50_ = 0.50 ± 0.07–5.05 ± 0.16 µM), with particular emphasis to derivative **36** (IC_50_ = 0.50 ± 0.07 µM). Due to its in vitro activity, the derivative was further evaluated against an animal model of *L. donovani* infection, being active through both oral and intramuscular administrations (IC_50_ = 0.80 ± 0.12 mg/kg body weight/mice/day and IC_50_ = 0.75 ± 0.20 mg/kg body weight/mice/day, respectively). From a mechanism of action perspective, the authors suggest that this compound’s activity is closely related to a critical interference in the parasite’s mechanism of defense against oxidative stress, particularly in the thiol redox pathway. In particular, it became clear that this molecule is capable of downregulating the expressions of the thiol-dependent enzymes transcriptionally and inhibiting the tyrosine reductase (TyrR) activity at a micromolar concentration. In addition to the inhibition of TyrR, this compound can also induce the host’s pro-inflammatory response, creating a dual effect against the intracellular parasite.

**Figure 11:**
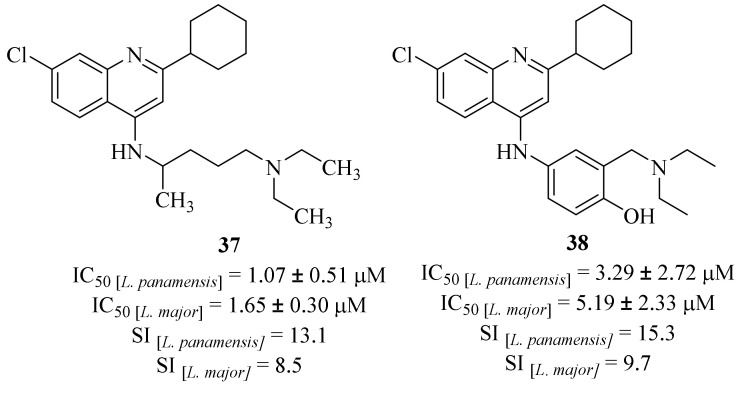
Structure of two promising antileishmanial compounds (**37** and **38**) against both *L. panamensis* and *L. major*.

A year later, in 2016, Herrera et al. evaluated the antileishmanial effects of bi- and tricyclic *N*-heterocycles against *L. panamensis* and *L. major* promastigotes and intracellular amastigotes, with particular emphasis on the quinoline derivatives evaluated [36]. Interestingly, these quinoline derivatives were inactive against the promastigote stage of both *Leishmania* species. However, two of the series of twenty evaluated quinolines (**37** and **38**, Figure 11) demonstrated promising activity levels against both *L. major* and *L. panamensis* intracellular amastigotes. In addition, both derivatives were more active against *L. panamensis* than *L. major*, with particular emphasis on derivative **37** for being the most active quinoline derivative (IC_50_ [*L. panamensis*] = 1.07 ± 0.51 µM and IC_50_ [*L. major*] = 1.65 ± 0.30 µM, Figure 11). Further studies showed that these derivatives inhibit the production of IL-10 by macrophages infected with *Leishmania*, suggesting that the compound-induced parasite-killing mechanism may be associated with the regulation of macrophage activation. Four years later, in 2020, the same research group evaluated derivative **37** in an animal model of *L. panamensis* infection, which corroborated this compound’s potential against Leishmania and the compound-induced inhibition of IL-10 production by macrophages [37].

**Figure 12:**
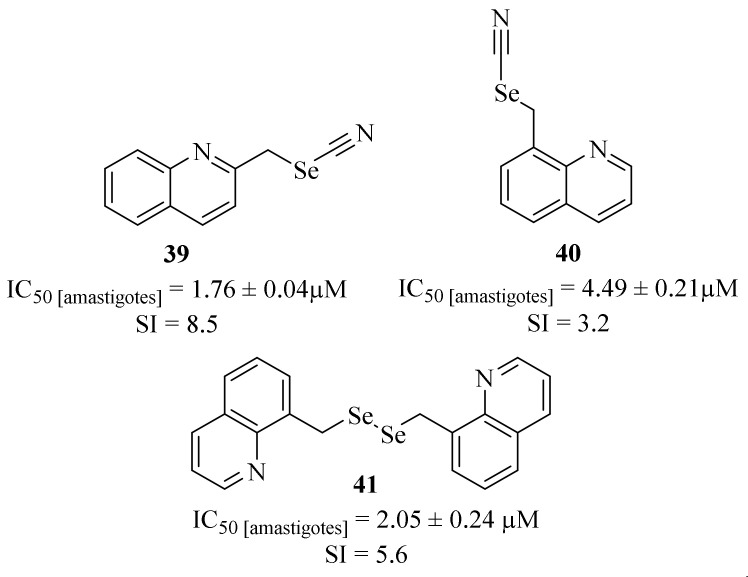
Quinoline-containing selenocyanates (**39** and **40**) and diselenides (**41**) with antileishmanial activity.

Still, in 2016, Baquedano et al. designed a series of new selenocyanates and diselenides containing several bioactive scaffolds as potential antileishmanial agents against *L. infantum* axenic amastigotes, from which it is important to emphasize three quinoline-containing derivatives (Figure 12) [38]. Their results demonstrated that, even with a scarce number of derivatives, the quinoline-containing compounds present promising antileishmanial properties in addition to low levels of toxicity against THP-1 cells. Furthermore, it was also possible to verify that the position of the selenyl substitution in the quinoline scaffold considerably affects the compound’s antileishmanial activity, with the 2-substituted quinoline (**39**) being significantly more active than the 8-substituted one (**40**) (IC_50_ = 4.49 ± 0.21 µM and 1.76 ± 0.04 µM, respectively). Finally, the dimerization of this type of compound can also promote significant improvements in terms of antileishmanial activity, with compound **41** (IC_50_ = 2.05 ± 0.24 µM) being two times more active than the corresponding monomer (**40**).

**Figure 13:**
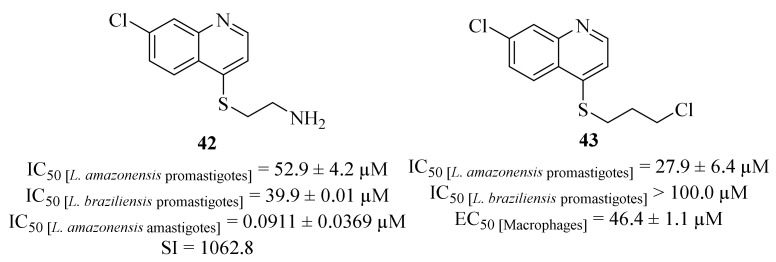
Promising 4-substituted quinolines (**42** and **43**) against *L. amazonensis* and *L. braziliensis*

Another research group focused their efforts on the synthesis and evaluation of a series of 4-substituted quinoline derivatives against *L. amazonensis* and *L. braziliensis* promastigotes and *L. amazonensis* amastigotes [39]. Following some of their previous works [40,41] this novel series of derivatives contemplated quinoline derivatives in which the two amino groups of the side chain were replaced by sulfur, hydroxy or chloro substituents. Considering their effects against the promastigote stage of both *L. amazonensis* and *L. braziliensis*, it was possible to verify that only two derivatives (**42** and **43**, Figure 13) present moderate to significant levels of antileishmanial activity against *L. amazonensis* (IC_50_ = 52.9 ± 4.2 µM and IC_50_ = 27.9 ± 6.4 µM, respectively), while only one of the derivatives (**42**) was active against *L. braziliensis* (IC_50_ = 39.9 ± 0.01 µM). However, derivative **43** also demonstrates high levels of toxicity against murine macrophages (IC_50_ = 46.4 ± 1.1 µM). In turn, against *L. amazonensis* amastigotes, derivative **42** was the only one to show considerable efficiency, with an IC_50_ = 0.0911 ± 0.0369 µM, being 139 times more active than the reference drug miltefosine (IC_50_ = 12.7 ± 0.9 µM). Further studies also allowed the authors to suggest that the antileishmanial effects of this derivative (**42**) might be associated with the induction of a high generation of ROS with low alterations of the mitochondrial membrane potential and without affecting plasma membrane, being mediated by mitochondrial oxidative stress.

**Figure 14:**
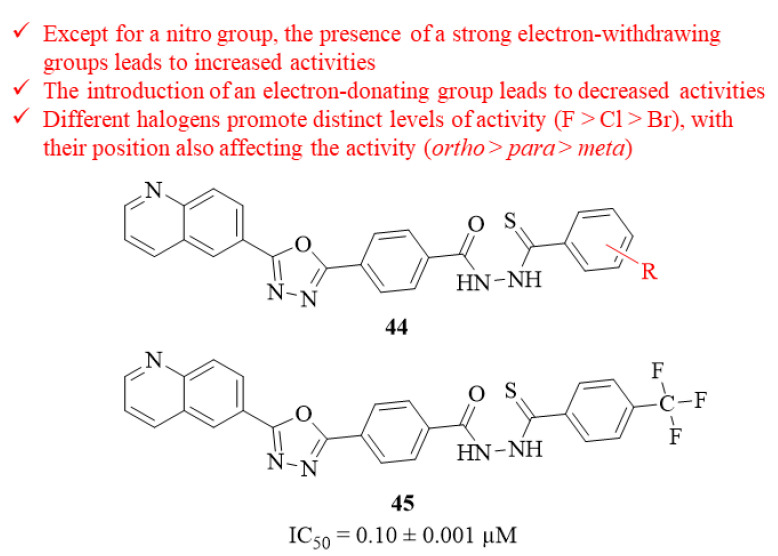
Structure-antileishmanial activity relationship study of quinolinyl-oxadiazole thiosemicarbazide hybrids (**44**), with emphasis on the most active derivative (**45**).

In the same year, a series of eighteen quinolinyl-oxadiazole thiosemicarbazide hybrids were designed and evaluated for its antileishmanial potential against *L. major* intracellular amastigotes by Taha et al. [42]. The results demonstrated that, from this series of eighteen hybrids, thirteen of them present significant levels antileishmanial activity (IC_50_ = 0.10 ± 0.001–7.40 ± 0.41 µM), being comparable with the reference drug, pentamidine (IC_50_ = 7.02 ± 0.09 µM). Structurally, it became clear that the presence of a strong electron-withdrawing group like the *p*-trifluoromethyl group (**45**, IC_50_ = 0.10 ± 0.001 µM) considerably contributes to the antileishmanial properties of this type of compound (Figure 14). However, the introduction of another type of electron-withdrawing group, like a nitro group, was also able to originate derivatives with poor antileishmanial activities (IC_50_ = 4.98 ± 0.21 µM–8.70 ± 0.30 µM) In turn, the introduction of an electron-donating group, like a methoxyl group, promoted the lowest levels of activity (IC_50_ = 7.40 ± 0.41 µM–18.12 ± 0.85 µM), suggesting that this type of functionalization might be detrimental to the antileishmanial properties of these compounds. Considering the halogenation pattern of the aromatic ring, one can assume that the presence of different halogen atoms promotes distinct levels of antileishmanial activity, with the fluoro substituted analogues being the most active derivatives (IC_50_ = 0.15 ± 0.001–3.30 ± 0.01 µM), followed by the chlorinated (IC_50_ = 0.72 ± 0.01–4.50 ± 0.15 µM) and the bromated ones (IC_50_ = 6.9 ± 0.20 µM–21.40 ± 0.50 µM). Interestingly, the presence of a simple methyl group in the aromatic ring also promoted high levels of activity (IC_50_ = 1.12 ± 0.01 µM), which might indicate that the introduction of alkyl chains would also be beneficial to the evaluated activity. Finally, the position of these halogens in the aromatic ring also affects its antileishmanial activity, with the *ortho*-position being the optimal introduction, followed by the *para*-position and the *meta*-position.

**Figure 15:**
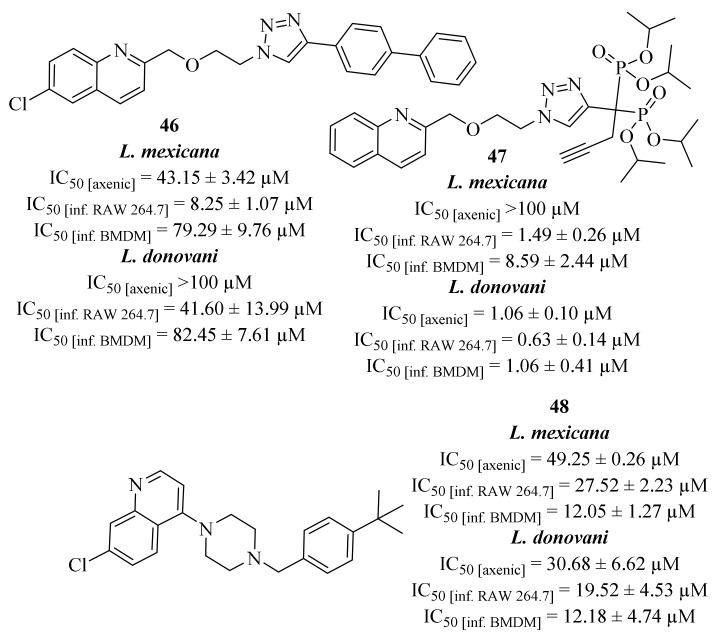
Quinoline-based GDP-MP competitive inhibitors (**46**–**48**) against *L. donovani* and *L. mexicana*.

Following some of their previous works in which some 2-substituted quinolines were already identified as antileishmanial leads, [27,43] Mao et al. performed a preliminary molecular study that allowed them to suggest that the quinoline motif could replace the guanine group of GDP-mannose within the GDP-MP catalytic site [44]. This evidence prompted them to design a series of one hundred GDP-MP competitive inhibitors, some containing the quinoline core in the inhibitor scaffold, and evaluate it against the pure enzyme (GDP-MP), from both *L. donovani* and *L. mexicana*, as well as against both parasite species. In a first evaluation, this series of derivatives was evaluated against two *Leishmania* and one human GDP-MPs, with only eleven derivatives demonstrating IC_50_ values below the screening concentration (100 µM). However, only five of them present considerable levels of affinity by exhibiting significant *Ki* values on a leishmanial GDP-MP, being three of them quinoline-containing derivatives (**46**–**48**, Figure 15). These three derivatives (**46**–**48**) were then evaluated against both axenic and intracellular amastigotes of *L. donovani* and *L. mexicana* on two cell host models, RAW264.7 macrophages and bone marrow derived macrophages (BMDM). Considering *L. donovani*, derivative **47** presents the most interesting levels of antileishmanial activity, in both axenic (IC_50_ = 1.06 ± 0.10 µM) and intracellular amastigotes (IC_50_ [RAW 264.7] = 0.63 ± 0.14 µM and IC_50_ [BMDM] = 1.06 ± 0.41 µM), while the remaining derivatives only demonstrate moderate levels of activity. In turn, considering *L. mexicana*, the three derivatives (**46**–**48**) demonstrate moderate to no activity against axenic amastigotes but significant levels of activity against intracellular amastigotes. In particular, derivative **46** demonstrates high levels of activity against infected RAW 264.7 macrophages (IC_50_ = 8.25 ± 1.07 µM), while derivative **48** only presents significant activity levels against infected BMDM (IC_50_ = 12.05 ± 1.27 µM). Once again, derivative **47** was the most active derivative against the intracellular amastigotes (IC_50_ [RAW 264.7] = 1.49 ± 0.26 µM and IC_50_ [BMDM] = 8.59 ± 2.44 µM), highlighting this derivative as the most promising compound for further developments.

**Figure 16:**
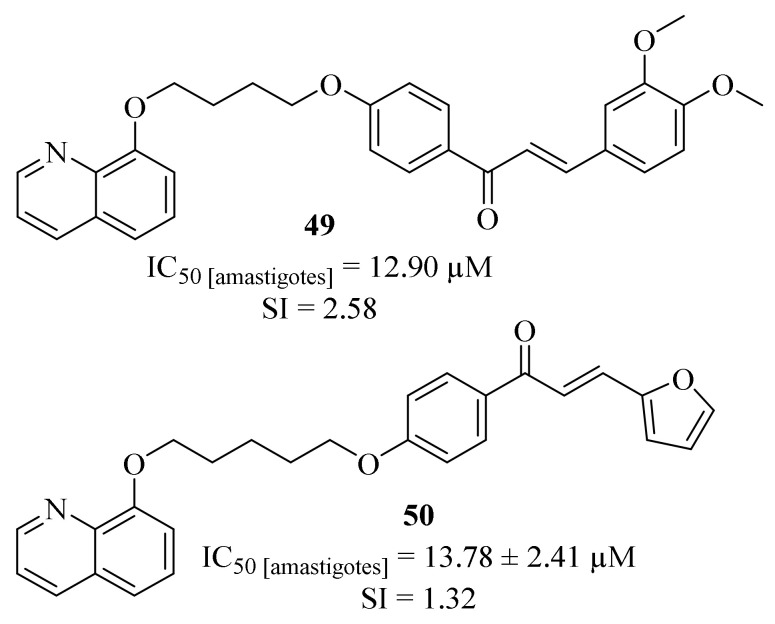
Structures of the most promising chalcone-quinoline (**49**) and furanchalcone–quinoline (**50**) hybrids against *L. panamensis*.

Still in 2017, Coa et al. designed a series of quinoline-chalcone and quinoline-chromone hybrids and evaluated their potential as antileishmanial agents against *L. (V) panamensis* amastigotes [45]. The results demonstrated that, from this series of eleven hybrids, four of them present significant levels of antileishmanial activity (EC_50_ < 20 µM), with particular emphasis to compound **49** (IC_50_ = 12.90 µM and SI = 2.58, Figure 16). Even though there is no clear relation between the antileishmanial activity and the length of the alkyl linker, it was still possible to verify that this hybridization approach promotes a combined improvement for this type of hybrids when compared with the corresponding parent compounds. In particular, through hybridization, this research group was able to design molecules that present higher activity levels than parent chromone (IC_50_ = 718.45 µM), while showing lower levels of cytotoxicity than the parent quinoline (IC_50_ = 1.38 µM).

In the beginning of 2018, and following the biological potential of molecular hybridization, the same research group focused their efforts on the development of a new series of furanchalcone–quinoline hybrids, amongst other types of hybrids, (Figure 16) and their evaluation against *L. (V) panamensis* intracellular amastigotes [46]. The results demonstrated that the newly synthesized furanchalcone–quinolines only present moderate levels of antileishmanial properties, with IC_50_ = 13.78 ± 2.41–207.36 ± 14.98 µM. Once again, relationship between the antileishmanial activity and the length of the alkyl linker was not clear, with the highest activity being achieved with a five-carbon alkyl chain (**50**, IC_50_ = 13.78 ± 2.41 µM), being consistent with the results obtained in the previous study.

**Figure 17:**
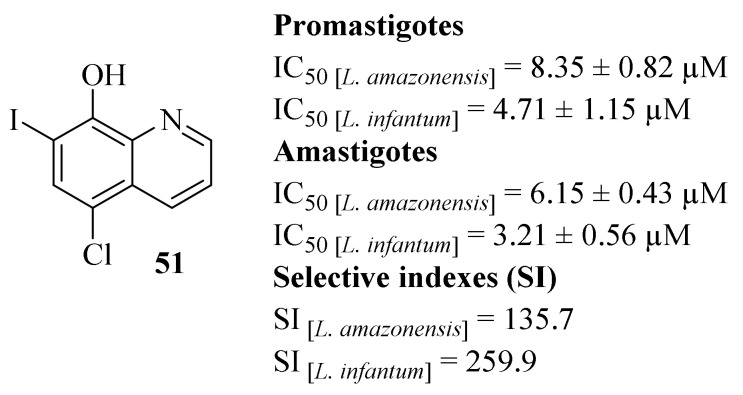
Clioquinol (**51**), a promising antileishmanial agent.

By the beginning of 2018, Tavares et al. decided to fully evaluate the compound 5-chloro-7-iodoquinolin-8-ol, also known as clioquinol (**51**, Figure 17) for its potential against *L. infantum* and *L. amazonensis* promastigotes and amastigotes, as well as its cytotoxicity effects against murine macrophages and human red cells [47]. Considering its effects on *Leishmania* promastigotes, clioquinol (**51**) demonstrates promising antileishmanial properties against both *Leishmania* species, with IC_50_ = 8.35 ± 0.82 µM and 4.71 ± 1.15 µM against *L. amazonensis* and *L. infantum*, respectively. In turn, regarding *Leishmania* axenic amastigotes, this compound also demonstrates high levels of activity, with IC_50_ = 6.15 ± 0.43 µM (*L. amazonensis*) and 3.21 ± 0.56 µM (*L. infantum*), being more active against this second stage of the parasite. Comparing with the reference drug amphotericin B (AmB), even though clioquinol (**51**) presents slightly lower levels of antileishmanial properties, this compound was considerably less toxic against murine macrophages, originating high SI values (135.7 for *L. amazonensis* and 259.9 for *L. infantum*). Furthermore, the compound’s mechanism of action was also evaluated against *L. amazonensis*, allowing us to observe that the treatment of promastigotes with clioquinol (**51**) induces changes in cell mobility and morphology. In particular, the effects promoted by the treatment with clioquinol (**51**) include a significant cell volume reduction, alterations in the mitochondrial membrane potential and also the induction of oxidative stress, culminating in the rupture of the plasma membrane. Finally, the toxicity of this compound was also evaluated in BALB/c mice demonstrating that the administration of clioquinol (**51**) presents no toxicity in this animal model, thus being considered safe for therapeutic usage and future developments for the treatment of *Leishmania*-infected hosts.

**Figure 18:**
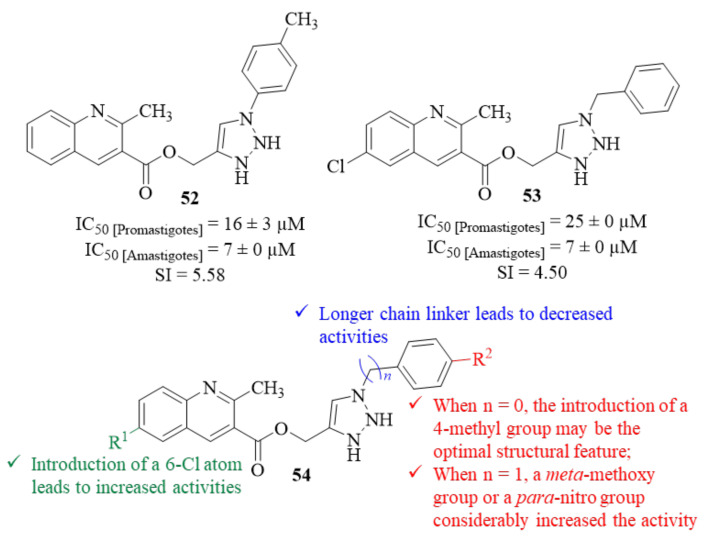
Structure-antileishmanial activity relationship study of quinoline-triazole hybrids (**54**), with particular emphasis to the most promising derivatives (**52** and **53**).

In the same year and based on previous reports on the antileishmanial effects of both quinoline and 1,2,3-triazole containing compounds, another research group followed the hybridization strategy to design a series of twenty-five quinoline-triazole hybrids [48]. These hybrids were then evaluated for their potential as antileishmanial agents against *L. donovani* promastigotes and amastigotes. Considering its effects on *Leishmania* promastigotes, the results demonstrated that this entire series present moderate to considerable antileishmanial activities (IC_50_ = 2.76–45.75 µM). However, regarding the intracellular amastigotes, only nine derivatives present considerable levels of antileishmanial activities, with particular emphasis on derivatives **52** and **53** (IC_50_ = 7 ± 0 µM, Figure 18) for presenting activity levels comparable to the reference drug miltefosine (IC_50_ = 8 ± 2 µM). Structurally, the results indicated that the distance between the triazole and phenol groups has a considerable influence on these derivatives’ antileishmanial properties (54, Figure 18). This suggestion is clearly corroborated by the fact that, for two derivatives with the same substitution pattern and only varying the linker length, the one with longer chain (*n* = 1, IC_50_ = 22 ± 0 µM) presents a considerable decrease of activity when compared with base derivative (*n* = 0, IC_50_ =7 ± 0 µM). Considering the substitution pattern introduced in phenol fragment, it was possible to verify that the introduction of a 4-methyl group may be the optimal structural feature for this type of activity for compounds with *n* = 0, originating the most active compound against *L. donovani* amastigotes (IC_50_ =7 ± 0 µM). However, when *n* = 1, the presence of this 4-methyl group seems to be less effective, leading to a significant decrease in activity (IC_50_ =34 ± 1 µM). In this case, the replacement of the 4-methyl group by both an electron donating group (3-methoxy group) at *meta*-position, or an electron-withdrawing group (4-nitro group) considerably increased the compound’s antileishmanial activity (IC_50_ = 22 ± 0 µM and IC_50_ = 18 ± 0 µM, respectively). Finally, the introduction of a 6-Cl atom in the quinoline fragment originates derivatives with higher antileishmanial activities than those without any substitution in this position. When evaluated in vivo by monitoring the parasite burden of a golden hamster’s spleen, only derivative **53** demonstrated promising activity against *L. donovani* intracellular amastigotes, presenting consistent levels of activity up to day 28 post-treatment (37.81 ± 10.46% [7th day] and 46.89 ± 4.26% [28th day]), with derivative **52** only demonstrating moderate levels of activity 28 days (40.36 ± 6.05% [28th day]).

**Figure 19:**
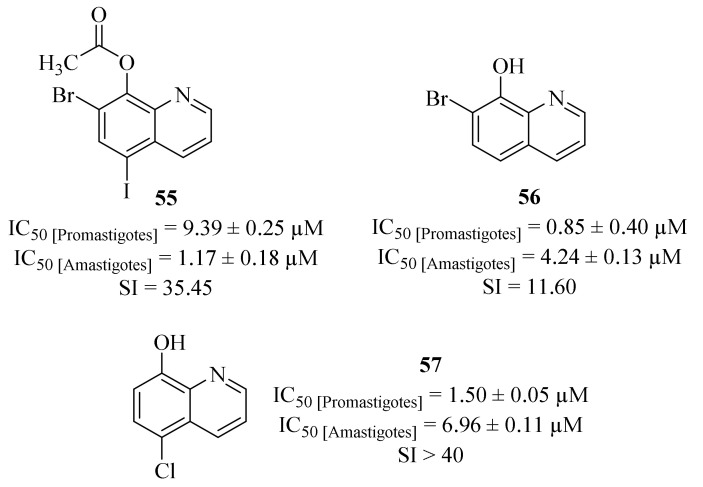
Most promising quinoline derivatives (**55**–**57**) against *L. (L.) amazonensis* amastigotes.

Another research group compiled a series of twenty-two quinoline derivatives, three commercially available and nineteen synthesized, and evaluated them against *L. (L.) amazonensis* promastigotes and amastigotes, particularly a strain capable of inducing anergic diffuse cutaneous leishmaniasis [49]. Considering its effects against *Leishmania* promastigotes, all the evaluated derivatives demonstrate significant levels of antileishmanial activity (IC_50_ < 10 µM), with seven of them being even more effective than the reference drug miltefosine (IC_50_ = 7.88 ± 2.11. µM). In turn, regarding their potential against *Leishmania* amastigotes, only eight derivatives were active against the intracellular stage of the parasite, presenting IC_50_ values ranging from 1.17 ± 0.18 µM to 29.62 ± 1.43 µM, with most of them being more active than miltefosine (IC_50_ = 31.36 ± 3.78 µM). From this series of derivatives, the most active compounds were derivatives **55** (IC_50_ = 1.17 ± 0.18 µM), **56** (IC_50_ = 4.24 ± 0.40 µM) and **57** (IC_50_ = 6.96 ± 0.11 µM), with particular emphasis to derivative **57** for additionally presenting the highest SI value (SI > 40, Figure 19). Unfortunately, the results obtained against *Leishmania* amastigotes were not conclusive enough to establish a proper structure-antileishmanial activity relationship study for this type of compounds.

**Figure 20:**
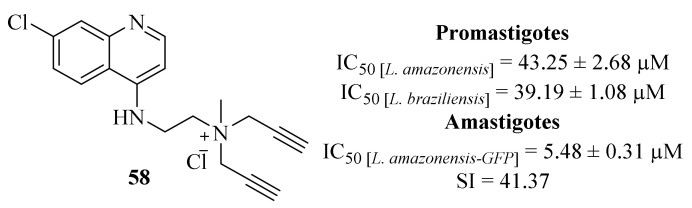
Most promising quinolinic salt (**58**) developed against *L. amazonensis* and *L. braziliensis*.

Still, in 2018, Calixto et al. synthesized a series of organic salts from active molecules, in a strategy intended to improve the biological and the physical-chemical properties of these compounds. Following a previous study in which some quinoline derivatives presented low levels of antileishmanial activities [41], despite being considerably active against other protozoans, this research group focused their efforts on derivatizing these compounds into organic salts to achieve higher levels of antileishmanial activity against *L. amazonensis* and *L. braziliensis* promastigotes and amastigotes (Figure 20) [50]. Considering its effects against promastigotes, the results demonstrated that only one derivative (**58**) is effective against both species of *Leishmania* (IC_50_ [*L. amazonensis*] = 43.25 ± 2.68 µM and IC_50_ [*L. brazilensis*] = 39.19 ± 1.08 µM). Furthermore, this derivative (**58**) was also the only active molecule against *L. amazonensis*-GFP intracellular amastigotes (IC_50_ = 5.48 ± 0.31 µM), a value similar to the observed against *L. amazonensis*-Wild type amastigotes (IC_50_ = 5.62 µM), while also presenting a low level of toxicity against murine macrophages (IC_50_ = 226.70 ± 0.31 µM). In terms of mechanism of action, this compound (**58**) induced a considerable reduction in the membrane potential and mitochondrial swelling, leading to its dysfunction and impairing the survival of the parasite. Furthermore, the treatment of promastigotes with this compound (**58**) promoted several morphological modifications such as rounded bodies and reduction of cell volume, alterations usually associated with apoptosis-like cell death. Finally, the treatment with this compound (**58**) also inhibits the formation of autophagic vacuoles while promoting the production of ROS, leading to an accelerated cell death.

**Figure 21:**
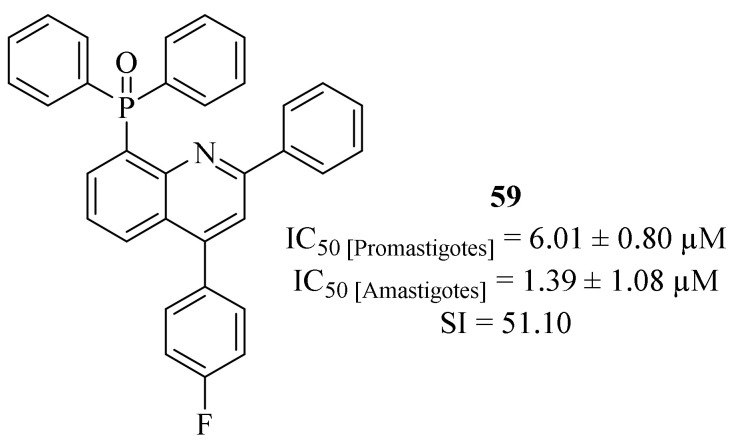
Structure of the most promising quinolinyl-phosphine oxide (**59**) against *L. infantum*.

By the end of 2018, Tejería et al. synthesized a series of five quinoline derivatives containing phosphorus substituents such as phosphine, phosphine sulfide and phosphine oxide groups, and evaluated them against *L. infantum* promastigotes and amastigotes [51]. The results demonstrated that the quinoline derivatives containing phosphine oxide groups were considerably more active against both stages of the parasite than the ones containing phosphine sulfide groups, with the latter group presenting non considerable values of activity against intracellular amastigotes (IC_50_ > 10 µM). The quinoline derivatives containing phosphine oxide groups presented significant levels of antileishmanial activity against both promastigotes (IC_50_ = 2.33 ± 0.25–6.01 ± 0.80 µM) and intracellular amastigotes (IC_50_ = 1.39 ± 1.08–4.14 ± 1.64 µM). In particular, it is important to highlight derivative **59** (Figure 21) for, not only being the most active quinoline derivative against intracellular amastigotes (IC_50_ = 1.39 ± 1.08 µM), but also for presenting the higher value of selectivity index (SI = 51.10).

**Figure 22:**
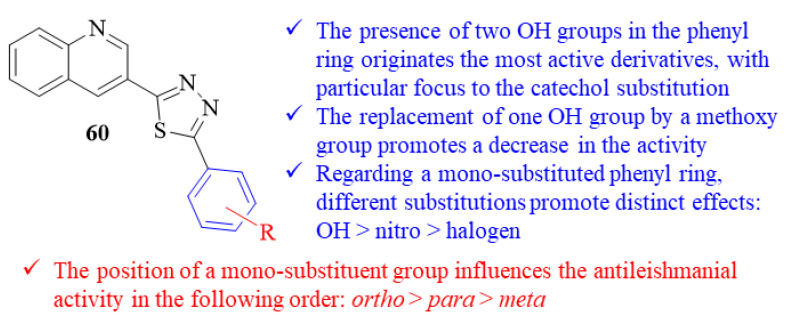
Structure-antileishmanial activity relationship study of quinoline-thiadiazole hybrids (**60**) against *L. major* intracellular amastigotes.

At the same time, another research group focused their efforts on designing a series of twenty quinoline-thiadiazole hybrids and evaluated them against *L. major* intracellular amastigotes [52]. From this series of twenty quinoline-thiadiazole hybrids, sixteen derivatives presented levels of antileishmanial activity comparable to the reference drug pentamidine (IC_50_ = 7.02 ± 0.09 µM), with IC_50_ values ranging from 0.04 ± 0.01 µM to 5.60 ± 0.21 µM. Structurally, the results demonstrated that the presence of two hydroxy groups in the phenyl ring originates the most active derivatives from the entire series (IC_50_ = 0.04 ± 0.01–0.90 ± 0.10 µM), with particular focus to the one bearing a catechol substitution (Figure 22). Furthermore, the replacement of one of these hydroxy groups by a methoxy group clearly promotes a decrease in the compounds’ antileishmanial activities (IC_50_ = 2.10 ± 0.10–4.10 ± 0.20 µM). When it comes to derivatives with a mono-substituted phenyl ring, the presence of a hydroxy group originates derivatives with higher antileishmanial activities (IC_50_ = 1.18 ± 0.10–3.40 ± 0.20 µM) than those bearing a nitro group (IC_50_ = 4.68 ± 0.20–8.20 ± 0.35 µM) or a halogen atom (IC_50_ = 0.98 ± 0.02–5.60 ± 0.21 µM). Interestingly, the position of these substitutions has a major influence on the compounds’ antileishmanial activities, with the results suggesting that the *ortho*-position plays a vital role in this activity followed by the *meta*- and *para*-positions.

**Figure 23:**
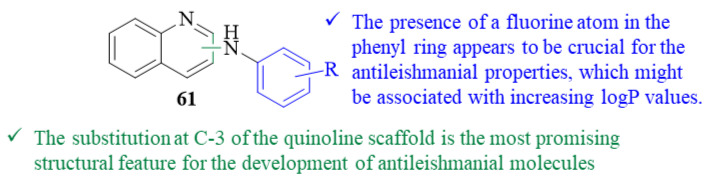
Structure-antileishmanial activity relationship study of aryl derivatives of 2- and 3-aminoquinoline (**61**) against *L. mexicana* promastigotes.

As part of a project intended to develop new safe chemotherapeutic agents against tropical diseases, Chanquia et al. synthesized a series of twelve aryl derivatives of 2- and 3-aminoquinoline and evaluated them for their antileishmanial potential against *L. mexicana* promastigotes (61, Figure 23) [53]. After 6 days of incubation, the results demonstrated that four derivatives show moderate levels of antileishmanial activity by inhibiting the growth of the parasite, with IC_50_ values ranging from 41.9 ± 0.8 µM to 98.1 ± 1.6 µM. The remaining eight derivatives showed no antileishmanial activity whatsoever, with IC_50_ > 200 µM. Structurally, the most active compounds were the ones bearing a fluorine atom in the phenyl ring, suggesting that the presence of this type of substitution might be crucial for these compounds’ antileishmanial properties. The authors suggest that this influence might be associated with the improved logP value promoted by the fluorine atom, which may facilitate cell membrane permeation. Furthermore, since three of the four active molecules consist in 3-aminoquinoline derivatives, one might assume that the substitution at C-3 of the quinoline scaffold is the most promising structural feature for the development of antileishmanial molecules.

**Figure 24:**
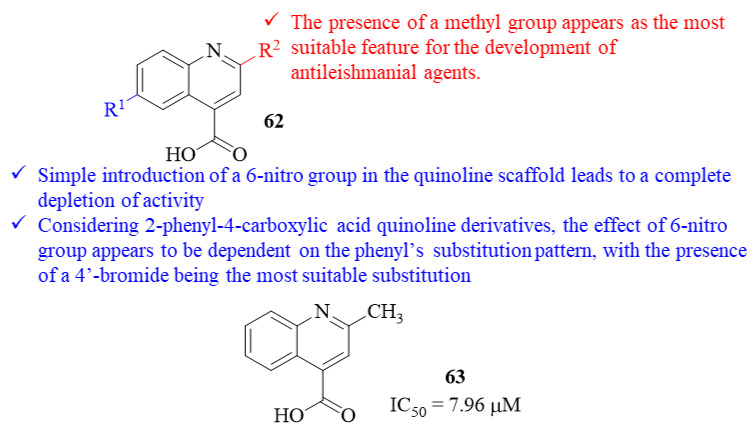
Structure-antileishmanial activity relationship study of quinoline-4-carboxylic acids (**62**) against *L. donovani* promastigotes, with emphasis on the most active compound (**63**).

In the beginning of 2019, Abdelwahid et al. synthesized a series of fifteen quinoline-4-carboxylic acids and evaluated them for its potential as antileishmanial agents against *L. donovani* promastigotes [54]. The results demonstrated that, from this entire series of derivatives, five derivatives present moderate to weak antileishmanial activities, with IC_50_ values ranging from 75.46 µM to 313.86 µM. Interestingly, one derivative (**63**, IC_50_ = 7.96 µM, Figure 24) emerged as being two times more potent than AmB (IC_50_ = 15.90 µM) and with an activity level comparable to the reference drug sodium stibogluconate (IC_50_ = 8.85 µM). Structurally, it was possible to verify that the simple introduction of a 6-nitro group in the quinoline scaffold leads to a complete depletion of the compound’s antileishmanial activity (IC_50_ = 7.96–925.93 µM). However, when considering 2-phenyl-4-carboxylic acid quinoline derivatives, the effect promoted by the 6-nitro group appears to be dependent on the substitution pattern on the phenyl fragment. In particular, in the presence of an EDG, like hydroxy or methoxy groups, the introduction of the 6-nitro group has a weak to no effect on the antileishmanial activity (IC_50_ = 791.65–683.28 µM and IC_50_ = 741.14–311.45 µM, respectively). In turn, in the presence of a 4′-bromide atom in the phenyl fragment, the same 6-nitro introduction promotes a significant improvement in the compound’s antileishmanial properties (IC_50_ = 313.86–46.07 µM). Also, in these 2-phenyl quinoline-4-carboxylic acid derivatives, the introduction of a 6-bromide atom has moderate to significant effects in the compounds’ antileishmanial activity (From IC_50_ = 313.86–741.14 µM to IC_50_ = 75.46–313.81 µM). Finally, when a naphthalene fragment at C-2 of the quinoline scaffold, the introduction of a 6-bromide atom leads to a considerable loss of activity (IC_50_ = 96.85–509.49 µM).

**Figure 25:**
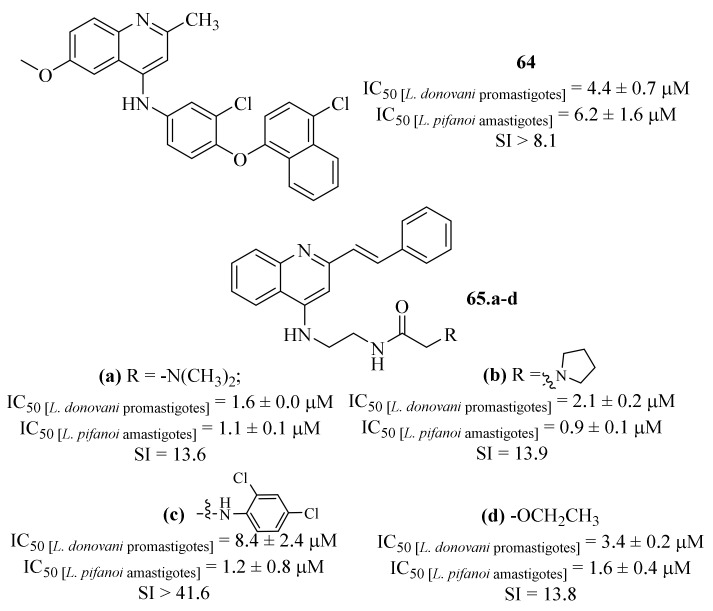
Structures of the most promising 4-aminostyrylquinolines (**65** and **66**) as antileishmanial agents against both *L. donovani* and *L. pifanoi*.

Based on the widely known potential of quinoline derivatives as antileishmanial agents, another research group designed a new class of 4-aminostyrylquinolines and evaluated them against *L. donovani* promastigotes and *L. pifanoi* amastigotes [55]. Furthermore, some other derivatives were also synthesized and evaluated to serve as a control group, allowing a deeper understanding of the 4-aminostyrylquinolines’ structural influences. Considering their effects against *L. donovani* promastigotes, the results demonstrated that, except for 2-styrylquinoline, all the evaluated molecules present considerable antileishmanial properties, with IC_50_ values ranging from 0.2 ± 0.0 µM to 35.1 ± 4.6 µM. Interestingly, most of the evaluated compounds were more potent than an already marketed antileishmanial quinoline (**64**, Figure 25) From this preliminary evaluation, it was already possible to verify the importance of a 2-styryl group since the removal of this particular structural feature leads to a considerable loss of antileishmanial activity (IC_50_ = 0.5 ± 0.1–10.9 ± 2.2 µM). Regarding the *Leishmania* amastigotes, once again most of the evaluated molecules present promising antileishmanial properties (IC_50_ = 0.9 ± 0.1–13.4 ± 3.8 µM), with particular emphasis to six derivatives that presented IC_50_ values below 1.5 µM. From these six most promising anti-amastigote derivatives, and considering their toxicity levels against J774 cells, four of them must be highlighted by presenting both promising antileishmanial properties and high levels of SI (**65.a**-**d**, Figure 25) Mechanistically, the authors demonstrated that these compounds’ antileishmanial activity is closely related with their effect on the parasite’s mitochondria, particularly by promoting mitochondrial dysfunction.

**Figure 26:**
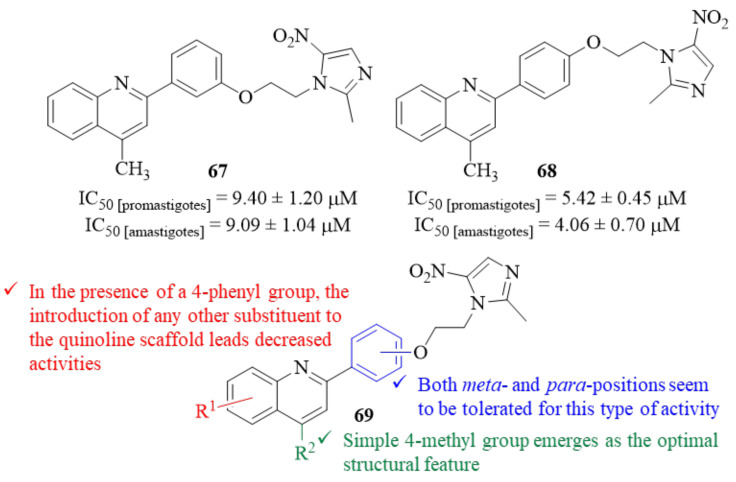
Structure -antileishmanial activity relationship study of quinoline-metronidazole hybrids (**69**), with emphasis on the most active derivatives (**67** and **68**).

Still, in 2019, and in an attempt to identify novel chemical scaffolds for the development of antileishmanial molecules, Upadhyay et al. developed a series of thirteen quinoline-metronidazole hybrids and evaluated them against *L. donovani* promastigotes and amastigotes [56]. The results demonstrated that, from this series of thirteen hybrids, only two derivatives (**67** and **68**, Figure 26) exhibit significant activity levels against both promastigotes and amastigotes in the preliminary screening at 50 µM and 25 µM. Based on this, the IC_50_ concentrations of these derivatives were also determined, with derivative **68** being the most active quinoline-metronidazole hybrid (IC_50 [promastigotes]_ = 5.30 ± 0.65 µM and IC_50 [amastigotes]_ = 4.06 ± 0.70 µM). Structurally, it was possible to verify that, regarding the substitution pattern in the quinoline fragment, a simple 4-methyl group emerges as the optimal structural feature for these compounds’ antileishmanial activity (**69**, Figure 26). Furthermore, in the presence of a 4-phenyl group, the introduction of any other substituent to the quinoline scaffold leads to a considerable decrease of activity. Regarding the hybridization position in the 2-phenyl group, both *meta*- and *para*-positions seem to be tolerated for this type of activity, originating compounds with similar activity levels (**67** and **68**). Focusing on the potential of these two derivatives (**67** and **68**), further in vivo evaluation was performed in a BALB/c model of VL, demonstrating that derivative **68** is much more effective in clearing parasite burden from both liver and spleen (>62% at 25 mg/kg) than derivative **67** (>50% at 50 mg/kg dose). Finally, in terms of mechanism of action, the authors demonstrated that derivative **68** is able to kill the parasite by inducing an apoptotic cascade, that begins with the disturbance of the mitochondria’s membrane potential, and also by promoting ROS and NO generation.

**Figure 27:**
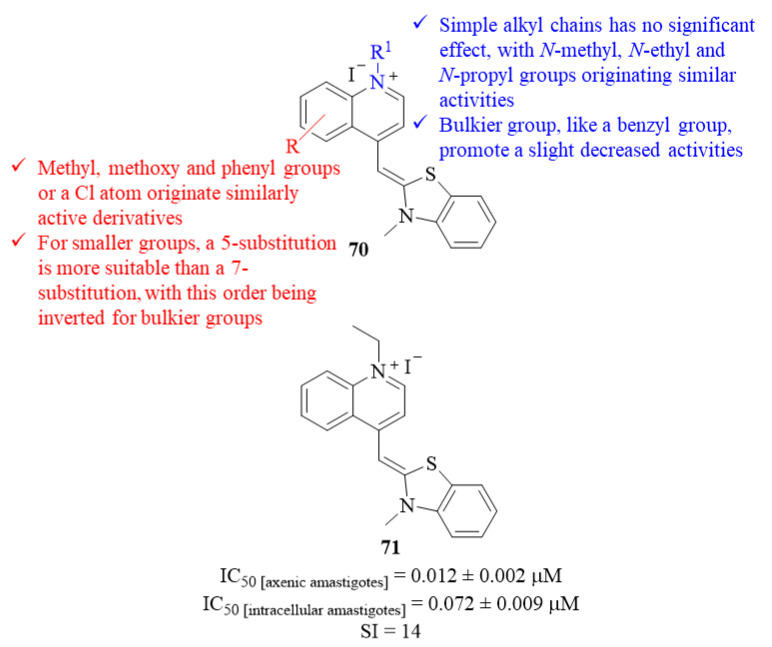
Structure-antileishmanial activity relationship study of thiazole orange analogs (**70**), with particular emphasis on the most active derivative (**71**).

Another research group, following the previously reported potential of cyanine compounds, focused their efforts on the development of a series of twenty-one thiazole orange analogs, and evaluated them against *L. donovani* axenic amastigotes [57]. The results demonstrated that these compounds are considerably active, presenting IC_50_ = 0.012 ± 0.002–0.042 ± 0.010 µM. Structurally, it was possible to verify that the introduction of simple alkyl chains in the nitrogen atom of the quinoline fragment has no significant effect on the compound’s antileishmanial activity, with *N*-methyl (IC_50_ = 0.014 ± 0.000 µM), *N*-ethyl (IC_50_ = 0.012 ± 0.002 µM) and *N*-propyl (IC_50_ = 0.013 ± 0.001 µM) groups originating derivatives with similar activities (**70**, Figure 27). In turn, the introduction of a bulkier group, like a benzyl group, promotes a slight decrease in the compound’s antileishmanial activity (IC_50_ = 0.026 ± 0.004 µM). While maintaining the *N*-methyl group, several modifications were also performed in the phenyl ring to understand the effects of different substituent groups and their position in the ring. The introduction of these different functional groups, such as methyl, methoxy and phenyl groups or a chlorine atom, had no considerable effect on the compounds’ antileishmanial properties originating derivatives with similar ranges of activity. However, it was still possible to understand that, for smaller groups, a 5-substitution originates the most active derivatives and a 7-substitution to the weaker derivatives, with this order being inverted for bulkier groups like a phenyl group. The relevance of both quinoline and benzothiazole fragments was assessed by replacing each of these fragments. These final modifications showed that replacing the benzothiazole fragment for a thiazole ring or the quinoline fragment for a pyridine ring leads to a considerable decrease in the compounds’ antileishmanial activities (IC_50_ = 0.014 ± 0.000–0.160 ± 0.010 µM and IC_50_ = 0.140 ± 0.030 µM, respectively). Finally, the most active compound (**71**, Figure 27) was also evaluated against *L. donovani* intracellular amastigotes presenting a lower but still considerable level of activity (IC_50_ = 0.072 ± 0.009 µM), being comparable to the activity presented by the reference drug AmB (IC_50_ = 0.045 ± 0.009 µM).

**Figure 28:**
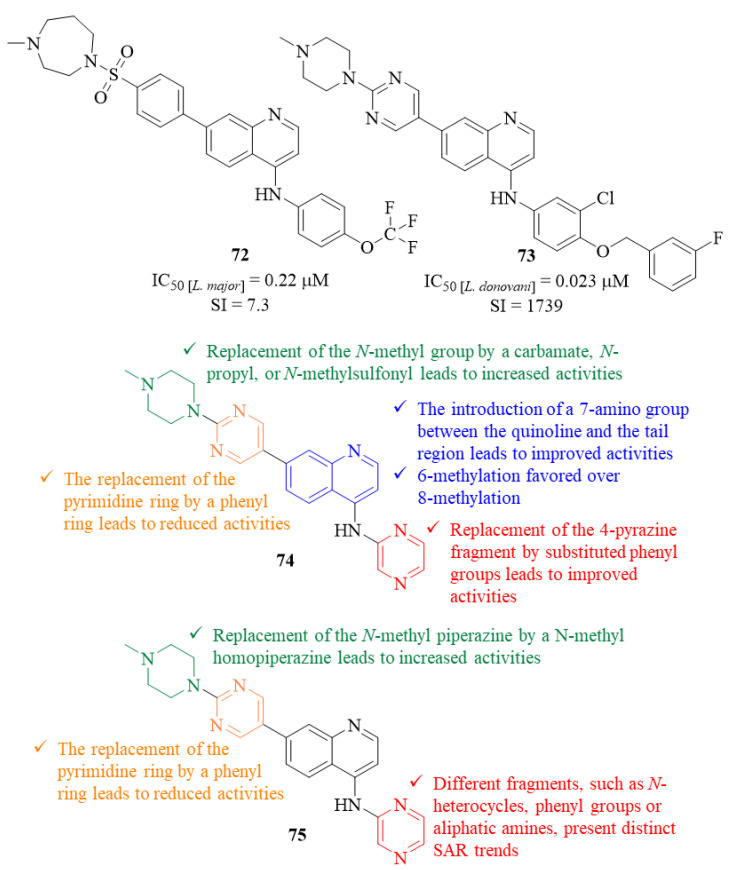
Structure-antileishmanial activity relationship studies of quinoline derivatives against *L. major* (**74**) and *L. donovani* (**75**), with particular emphasis on the most active quinoline against each species (**72** for *L. major* and **73** for *L. donovani*).

In the beginning of 2020, and following a target repurposing and parasite-hopping approach, Singh et al. developed a series of quinoline derivatives originated from the quinoline derivatives originated from the reoptimization of lapatinib to NEU-1953 and further optimizations of the latter [58,59,60]. This entire series of quinoline derivatives was then evaluated for its antileishmanial potential against *L. major* and *L. donovani* intracellular amastigotes [61]. Considering the effects against *L. major*, several derivatives present considerable levels of antileishmanial activity, with particular emphasis on the most active derivative (**72**, Figure 28) that exhibited an IC_50_ = 0.22 µM and a SI of 7.3. Structurally, for compounds containing a 4-pyrazine, the introduction of a 7-amino group between the quinoline and the tail region originates derivatives with significantly improved activities (IC_50_ > 15–1.7-5.3 µM). Furthermore, in the tail region, the replacement of the pyrimidine ring by a phenyl ring leads to a reduced level of antileishmanial activity (IC_50_ = 1.7–7.9 µM). Additionally, the replacement of the terminal *N*-methyl group by a carbamate (IC_50_ = 0.35 µM), a *N*-propyl (IC_50_ = 4.3 µM), or *N*-methylsulfonyl (IC_50_ = 0.73 µM) originates a significant increase of these compounds’ antileishmanial activities. Interestingly, the methylation of the quinoline fragment provides different effects depending on the position in which the methyl group was introduced, with a 6-methylation resulting in an improved activity (IC_50_ = 1.5 µM) and an 8-methylation originating a complete loss of activity (IC_50_ > 24 µM). Finally, the replacement of the 4-pyrazine fragment by substituted anilines resulted in derivatives with improved antileishmanial activities, particularly 3′-chloro-4′-methoxyaniline (IC_50_ = 1.6 µM) and 4-(trifluoromethoxy)aniline (IC_50_ = 0.22 µM).

Following the positive hits provided by the evaluation against *L. major*, a selection of analogs was further evaluated against *L. donovani*, leading to distinct activity trends. The results demonstrated that this additional evaluation identified three molecules with low micromolar inhibition (IC_50_ < 10 µM) and four with submicromolar activity levels (IC_50_ < 1 µM), with particular emphasis on derivative **73** that presents an IC_50_ = 0.023 µM and a SI of 1739. Structurally, considering the head fragment, the replacement of the 4-pyrazine ring by a tetrahydropyran originates a derivative with a significantly improved activity (IC_50_ > 15–4.0 µM). Furthermore, the combination of two distinct modifications, in this case the replacement of the piperazine with the homopiperazine and the pyrazine with 4-(trifluoromethoxy)aniline, led to the formation of one of the most active compounds from this series (IC_50_ = 0.085 µM).

**Figure 29:**
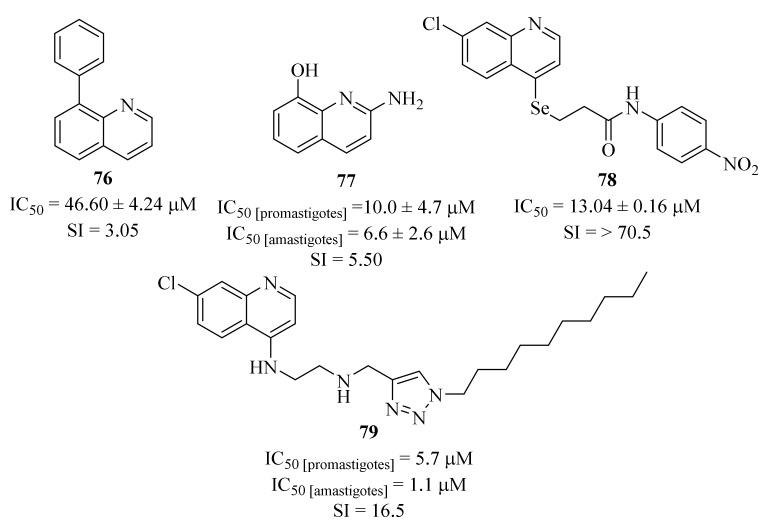
Promising quinoline derivatives against *L. (V) panamensis* (**76** and **77**) and *L. amazonensis* (**78** and **79**).

In the same year, another research group developed a series of eleven quinoline-biphenyl hybrids in the search for new therapeutic alternatives for the treatment of cutaneous leishmaniasis and evaluated them against *L. (V) panamensis* intracellular amastigotes [62]. The results demonstrated that, from the eleven derivatives, only five were moderately active against intracellular amastigotes (IC_50_ < 50 µM), with particular emphasis on derivative **76** (Figure 29) that presents an antileishmanial activity comparable with the reference drug meglumine antimoniate (IC_50_ = 46.60 ± 4.24 µM and 25.69 ± 5.74 µM, respectively). Structurally, it was possible to verify that the introduction of any substitution pattern in the phenyl ring would lead to a considerable decrease in the antileishmanial activity. Nevertheless, one could still assess that the effect promoted by the introduction of a methyl group is most accentuated that the one induced by a hydroxy group.

By the end of 2020, Suarez et al. used in silico techniques to identify synthetic quinoline alkaloids with a structure similar to the natural product *N*-methyl-8-methoxyflindersine and evaluated them against *L. (V.) panamensis* promastigotes and amastigotes [63]. The results demonstrated that, from the entire list of evaluated derivatives, only one (**77**, Figure 29) presents considerable levels of antileishmanial activity (IC_50 [promastigotes]_ = 10.0 ± 4.7 µM and IC_50 [amastigotes]_ = 6.6 ± 2.6 µM) and low levels of toxicity (SI = 5.5). This compound was further evaluated in an animal model of *L. (V.) panamensis* infection, demonstrating a 50% cure rate in addition to neutrophil and macrophage migration. In terms of mechanism of action, this compound is able to induce cell apoptosis in both promastigotes and intracellular amastigotes, leading to the inhibition of parasitic growth and development.

In 2021, and following their previous success in identifying new candidates for antileishmanial drug development, Huang et al. performed a virtual screening on a series of selenide-derived quinoline derivatives, synthesized the most likely to be active and evaluated them against *L. amazonensis* [64]. The biological evaluation of these compounds corroborates the prediction of the virtual screening, with the derivatives predicted to be active demonstrating promising antileishmanial activity levels (IC_50_ = 13.04 ± 0.16–171.11 ± 14.10 µM), with particular emphasis to derivative **78** (IC_50_ = 13.04 ± 0.16 µM).

In the same year, another research group focused their efforts on the synthesis of a novel series of quinoline-triazole hybrids and evaluated them against *L. amazonensis* promastigotes and intracellular amastigotes [65]. Considering both promastigotes and amastigotes, the results demonstrated that only one derivative (**79**, Figure 29) presents promising antileishmanial properties with an IC_50_ values of 5.7 µM and 1.1 µM against promastigotes and amastigotes, respectively. In terms of mechanism of action, this quinoline derivative exerts its antileishmanial effects by inducing a pronounced reduction of the mitochondrial membrane potential, leading to the disruption of its function. Furthermore, this disruption accelerates the generation of ROS and can also culminate in the collapse of the bioenergetic metabolism of the parasite. In conclusion, this derivative induces modifications to biochemical processes through the interference in the bioenergetic system and plasma membrane permeabilization, with consequent activation of apoptosis-like and necrosis processes, culminating in cell death.

**Figure 30:**
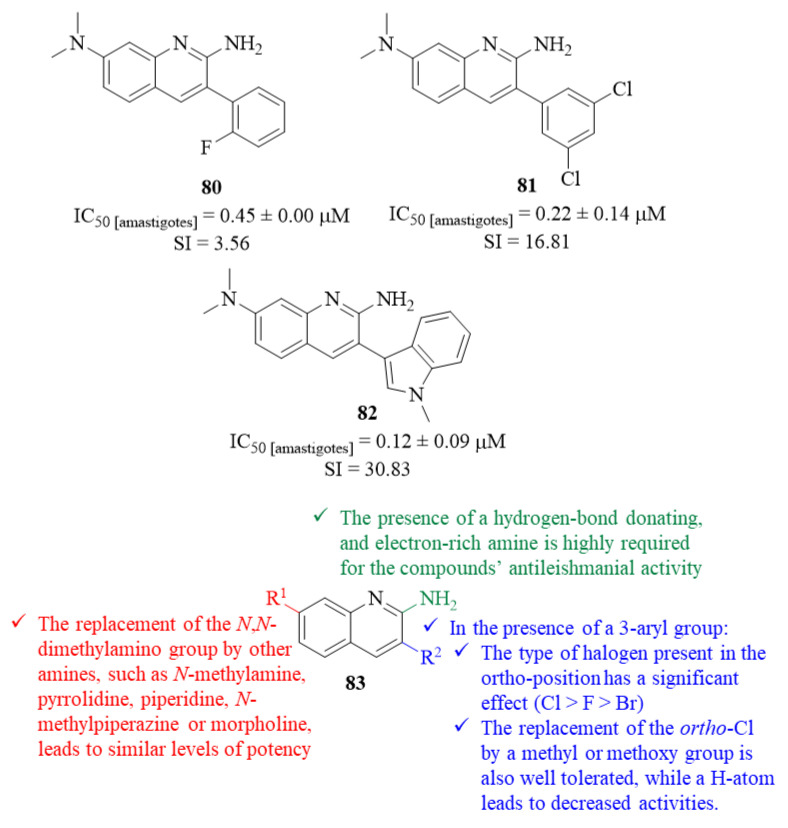
Structure-antileishmanial activity relationship study of 3-arylquinolines (**83**), with focus on the most active derivatives (**80**–**82**).

Hammill et al. continued their previous work by developing a novel series of 3-arylquinoline derivatives and evaluating them for their antileishmanial potential against *L. mexicana* intracellular amastigotes [66]. In this work, a phenotypic high-throughput screening was performed to identify novel antileishmanial leads, with more than 100 molecules being evaluated, leading to further dose–response assays for the most promising antileishmanial agents. To develop the most complete SAR study possible, this research group adopted a strategy based on the modification of four specific structural features, namely the importance of the quinoline scaffold itself and of the substituent group introduced at C-2 and C-7 positions and 3-aryl group, in comparison with derivative **80** (Figure 30).

Considering the quinoline scaffold, the results demonstrated that the quinoline fragment is crucial for the compound’s antileishmanial activity since the introduction of additional nitrogen atoms into the quinoline scaffold, its replacement with other bicyclic heterocycles and its fusion to an additional heterocyclic ring originates weaker derivatives (IC_50_ > 1.0 µM) than **80** (IC_50_ = 0.45 ± 0.00 µM). Maintaining the same quinoline scaffold fragment, a series of modifications were performed at C-2 to provide a deeper understanding about the optimal structural features to introduce to this quinoline position. From these modifications, one can verify that the replacement of the 2-amino group by any other structural feature originates weaker derivatives (IC_50_ > 1.0 µM), suggesting that the presence of a hydrogen-bond donating and electron-rich amine at this position is highly required for the compounds’ antileishmanial properties (Figure 30).

Regarding the influence of the 3-aryl group, while maintaining a 2-amino group, the results demonstrated that the type of halogen present in the *ortho*-position has a significant effect on the compounds’ antileishmanial properties, with an *ortho*-Cl atom originating the most active derivative (IC_50_ = 0.28 ± 0.07 µM), followed by *ortho*-F (IC_50_ = 0.45 ± 0.00 µM) and *ortho*-Br (IC_50_ = 0.61 ± 0.02 µM). However, the position of this Cl atom in the aryl ring seems to not affect the compounds’ antileishmanial properties, with all the derivatives presenting similar levels of activity (IC_50_ = 0.36 ± 0.04 µM for *meta*-Cl and IC_50_ = 0.40 ± 0.03 µM for *para*-Cl). In turn, the introduction of a second Cl atom promotes a wider range of antileishmanial activity levels, IC_50_ = 0.22 ± 0.14–0.76 ± 0.09 µM, with the derivative containing two *meta*-Cl atoms being the most active compound from this group (**81**, IC_50_ = 0.22 ± 0.14 µM, SI = 17). The replacement of the *ortho*-Cl by a methyl or methoxy group does not affect the antileishmanial properties (IC_50_ = 0.33 ± 0.02–0.30 ± 0.09 µM, respectively), while a hydrogen atom originates a considerably less active derivative (EC_50_ = 0.63 ± 0.17 µM). Based on the concerns that derivative **81** could have chemical properties that might limit its in vivo potential (fairly high hydrophobicity with cLogP = 4.86), the 3- dichlorophenyl ring was also replaced by a series of different heterocycles in an attempt to improve the compound’s chemical properties while retaining potency and selectivity. This optimization demonstrated that compounds containing five-membered heterocycles were considerably less effective (IC_50_ > 1.00 µM), while isosteric six membered heterocycles originate derivatives with similar antileishmanial activity levels (IC_50_ = 0.33 ± 0.03–0.87 ± 0.17 µM). The presence of more sterically encumbered bicyclic heterocycles was also well tolerated (IC_50_ = 0.12 ± 0.09–0.90 ± 0.84 µM), with derivative **82** being the most active from the group (IC_50_ = 0.12 ± 0.09 µM), suggesting that the potential target presents a deep and flexible hydrophobic pocket in this region (Figure 30).

Finally, while maintaining either the 3,5-dicholorophenyl ring or the *N*-methyl indole fragment, some modifications were also performed at 7-(*N*,*N*-dimethylamino) group to fully understand the steric and electronic tolerances of this specific position. The results demonstrated that the replacement of the *N*,*N*-dimethylamino group by other amines, such as *N*-methylamine, pyrrolidine, piperidine, *N*-methylpiperazine or morpholine, originates derivatives with similar levels of potency (IC_50_ = 0.37 ± 0.06–0.71 ± 0.33 µM for the 3,5-dicholorophenyl ring and IC_50_ = 0.16 ± 0.28–0.64 ± 0.15 µM for the *N*-methyl indole fragment). This evidence reveals that it is possible to modify this position in order to improve the physicochemical properties while retaining both potency and selectivity.

**Figure 31:**
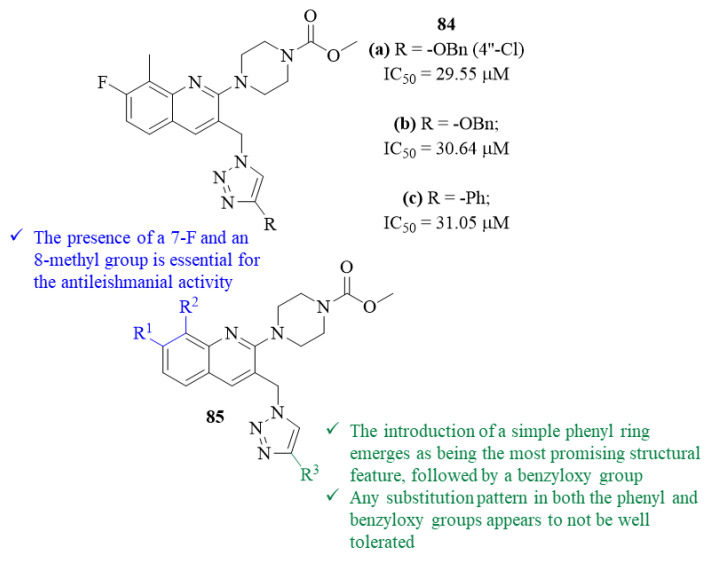
Structure-antileishmanial activity relationship study of quinoline-1,2,3-triazole hybrids (**85**), with emphasis on the most active derivatives from the series (**84**).

Still, in 2021, another research group developed a series of twelve novel quinoline-1,2,3-triazole hybrids and evaluated them against *L. donovani* promastigotes, followed by molecular docking studies against *L. major* pteridine reductase (*Lm*-PTR1) [67]. From their results, this research group was able to verify that three of the derivatives present significant levels of antileishmanial activity, with IC_50_ values ranging from 29.55 µM to 31.05 µM, similar to the reference drug AmB. Structurally, the results also indicated that the substitution pattern on the quinoline scaffold has a considerable effect on the compounds’ antileishmanial properties. In particular, it was possible to verify that the presence of a 7-F and an 8-methyl group is essential for the compounds’ antileishmanial activity, with the three most active derivatives containing this substitution pattern (Figure 31). Furthermore, the functional group introduced at C-4′ of the triazole ring also seems to significantly affect the antileishmanial properties of this type of compounds. In this case, the introduction of a 4″-chloro-benzyloxy group emerges as being the most promising structural feature, originating the most active compound from this series (**84.a**, IC_50_ = 29.55 µM), followed by the introduction of an unsubstituted benzyloxy group (**84.b**, IC_50_ = 30.64 µM) and a simple phenyl ring (**84.c**, IC_50_ = 31.05 µM). Except for a 4″-Cl atom in the benzyloxy group, the presence of any substitution pattern in both the phenyl and benzyloxy groups appears to not be well tolerated, originating derivatives with low to no activity whatsoever. In terms of mechanism of action, based on the molecular docking studies, the authors suggest that this might be associated with their effect on the enzyme PTR1, since all the derivatives demonstrate a significant binding affinity to the enzyme, with the theoretical results following the same pattern as the experimental ones.

**Figure 32:**
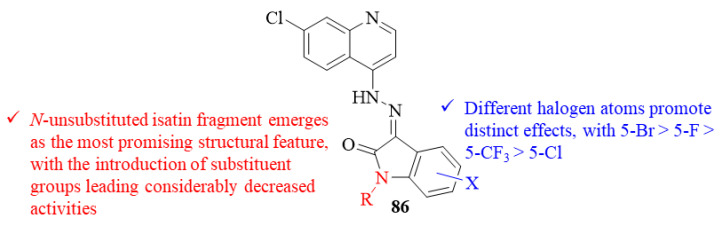
Structure-antileishmanial activity relationship study of quinoline-isatin hybrids (**86**) against *L. major*.

One year later, in 2022, Sabt et al. developed a series of twenty quinoline-isatin hybrids and evaluated them for their antileishmanial activity against both *L. major* promastigotes and amastigotes (Figure 32) [67]. Considering their activity against promastigotes, the results demonstrated that all the evaluated derivatives present considerable levels of antileishmanial activity with IC_50_ values ranging from 0.51 ± 0.06 µM to 5.95 ± 0.28 µM, being more active than the reference drug miltefosine (IC_50_ = 7.90 ± 0.26 µM). Structurally, it was possible to verify that the derivatives containing a *N*-unsubstituted isatin fragment constitute the group of most active derivatives from this series, IC_50_ = 0.51 ± 0.06–1.10 ± 0.14 µM, being more active than the parent hydrazine (IC_50_ = 3.00 ± 0.34 µM). In turn, the introduction of substituent groups at *N*-position leads to a considerable decrease in the compounds’ antileishmanial activities, when compared to their unsubstituted congeners (IC_50_ = 1.74 ± 0.12–5.95 ± 0.28 µM). The reduction of antileishmanial activity promoted by the *N*-substitution of the isatin core is also dependent on the functional group introduced, with a benzyl group causing a more accentuated decrease, while alkyl groups promote only a slight reduction. Finally, the substitution pattern of the *N*-unsubstituted isatin core also seems to have a significant effect on the compounds’ antileishmanial potential, with a 5-Br originating the most active derivative (IC_50_ = 0.51 ± 0.34 µM) followed by 5-F (IC_50_ = 0.56 ± 0.22 µM), 5-CF_3_ (IC_50_ = 0.67 ± 0.34 µM) and 5-Cl (IC_50_ = 0.67 ± 0.16 µM). Considering *L. major* amastigotes, the results showed that, except for one derivative, all the evaluated compounds were more active against this stage of the parasite than miltefosine (IC_50_ = 8.08 ± 0.22 µM), with IC_50_ values ranging from 0.60 ± 0.04 µM to 8.29 ± 0.32 µM. Structurally, the *N*-substitution of the isatin core promotes the same type of effect as observed against the promastigote stage, a decrease of the compounds’ antileishmanial activities, with the *N*-unsubstituted derivatives being the most active compounds against amastigotes (IC_50_ = 0.60 ± 0.04–2.45 ± 0.28 µM). This evidence, observed against both stages of the parasite, suggests that the presence of a group with the ability to form H-bonding interactions might be crucial for these compounds’ antileishmanial properties. In terms of mechanism of action, this research group evaluated the effects of the most active compounds against parasites supplemented with folic and folinic acids to assess if these compounds were acting against the parasite’s folate pathway. By adding folic and folinic acids, it was possible to verify the almost complete depletion of antileishmanial activity, similar to what happens with a known Lm-PTR1 inhibitor (trimethoprim). This fact confirms the anti-folate mechanism of this type of molecules through the inhibition of DHFR-TS and PTR1.

**Figure 33:**
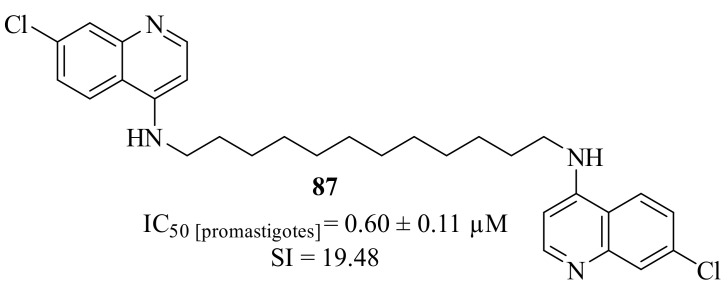
Structure of a quinoline dimer with promising antileishmanial properties against *L. infantum* promastigotes.

In the beginning of 2023, Silva et al. developed a series of 1,2,3,4-tetrahydroacridines, based on a virtual screening performed against the enzyme *S*-adenosylmethionine decarboxylase, and evaluated them against *L. infantum* promastigotes [68]. From their results, a SAR study was structured and it was possible to verify that the length of the alkyl chain has a significant effect on the compounds’ antileishmanial activity, especially in the case of dimers. Based on this evidence, and in the fact that this series of 1,2,3,4-tetrahydroacridines present high levels of toxicity, this research group decided to replace the tetrahydroacridine scaffold by a 7-chloroquinoline core. This replacement allowed the retention of the promising antileishmanial properties while considerably decreasing the compound’s toxicity, resulting in derivative **87** (Figure 33).

**Figure 34:**
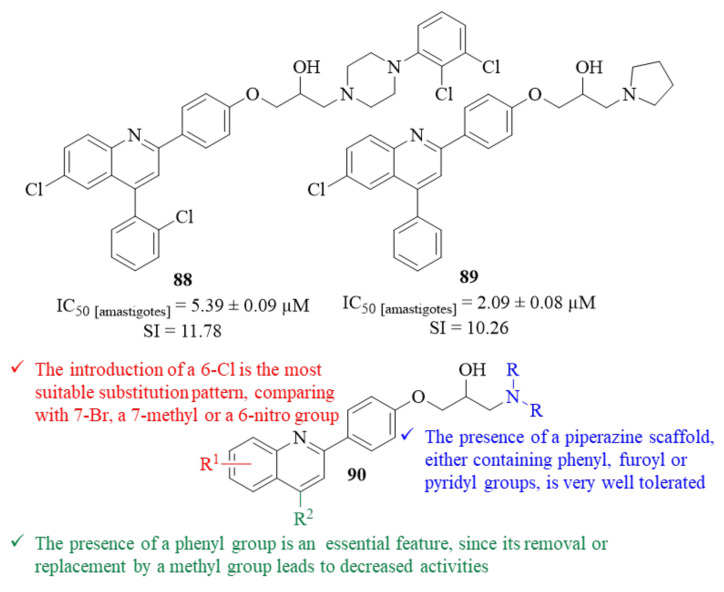
Structure-antileishmanial activity relationship study of quinoline-piperazine/pyrrolidine hybrids against *L. donovani* (**90**), with emphasis on the most promising derivatives (**88** and **89**).

By the end of 2023, another research group synthesized a series of thirty-five quinoline-piperazine/pyrrolidine hybrids and evaluated them against *L. donovani* intracellular amastigotes [69]. This work was based on the hypothesis that, by conjugating the quinoline moiety with piperazine/pyrrolidine scaffold, one might be able to design effective antileishmanial agents, considering the widely known potential of quinoline derivatives. The results demonstrated that, from this series of thirty-five hybrids, only twelve molecules present moderate to significant levels of antileishmanial activity, with IC_50_ values ranging from 2.09 ± 0.08 µM to 8.90 ± 0.18 µM. This series of hybrids can be divided into two distinct groups, twenty-four quinoline-piperazine derivatives and seven quinoline-pyrrolidine derivatives. Considering the first group, six derivatives emerged as being active against the amastigotes, IC_50_ = 5.39 ± 0.06–8.22 ± 0.15 µM. Structurally, it was possible to verify that the introduction of a 6-Cl atom into the quinoline scaffold is the most suitable substitution pattern, comparing with compounds containing 7-Br atom, a 7-methyl or a 6-nitro group (Figure 34). In addition, this introduction is not only responsible for retaining or increasing the compound’s antileishmanial activity but also for decreasing their toxicity, originating derivatives with improved SI values. Furthermore, regarding the C-4 of the quinoline scaffold, it became clear that the presence of a phenyl group is essential for the compounds’ antileishmanial properties, since its removal or replacement by a methyl group leads to inactive molecules at the evaluated concentrations. Finally, the presence of a piperazine scaffold as the terminal amino fragment, either containing phenyl, furoyl or pyridyl groups, is very well tolerated, leading to derivatives with moderate antileishmanial properties. In terms of the second group, the results demonstrated that five derivatives display moderate to significant levels of antileishmanial activity, with IC_50_ values ranging from 2.09 ± 0.08 µM to 8.89 ± 0.18 µM. Structurally, the introduction of a 6-Cl atom into the quinoline scaffold, similar to the first group, leads to an increase in the compounds’ antileishmanial activity while decreasing their toxicity (Figure 34). Following the promising in vitro results of derivatives **88** (IC_50_ = 5.39 ± 0.09 µM, SI = 11.78) and **89** (IC_50_ = 2.09 ± 0.09 µM, SI = 10.26), these compounds were selected for further in vivo antileishmanial evaluation. In this in vivo assay against *L. donovani* infected hamsters, both compounds continued to present significant levels of antileishmanial activity, with particular emphasis on derivative **88** for being able to considerably inhibit the *leishmania* parasite burden on the hamsters’ spleen (56.32 ± 4.23% at 50 mg/kg).

## 3. Discussion and Conclusions

As mentioned throughout this paper, until this day, the lack of an effective treatment against leishmaniasis remains one of the main concerns for the WHO, with organoantimonial drugs continuing to be the primary approach to treating this disease. Even though some other antileishmanial drugs have been developed throughout the years, such as amphotericin B, miltefosine, paromomycin, sitamaquine, and pentamidine, the associated side effects in addition to the parasite’s resistance mechanisms, have impaired the accomplishment of WHO’s goal to eradicate leishmaniasis in the near future. Thus, numerous research groups have focused on drug discovery and development programs to develop novel potential antileishmanial treatments.

Following this goal, many families of compounds have been evaluated for their antiprotozoal potential, particularly against *Leishmania*, leading to considerable improvements in the discovery and development of novel antileishmanial agents. These improvements have also inspired several review papers intending to compile the extensive information crucial for further research programs in the medical chemistry field [14,20,70]. As the family of quinoline derivatives is one of the most studied against *Leishmania*, this review was designed to provide a structural guideline for quinoline derivatives, which we believe to be critical for future developments in this field. Furthermore, this paper again proves the considerable potential of quinolines to be used against parasitic diseases, with particular emphasis on leishmaniasis.

Considering the information represented here, some structural features emerge as crucial for developing quinoline-based antileishmanial agents, commonly appearing in a wide range of derivatives. However, due to the virtually unlimited chemical space for structural, establishing a work-for-all guideline becomes almost impossible, with different substitution patterns on the quinoline scaffold leading to distinct levels of antileishmanial activity. Nevertheless, some structural functionalizations can be suggested to serve as a starting point for future drug discovery programs. In particular, it became clear that the presence of a positive charge in the quinoline derivatives, mainly in the form of a quinolinium salt, maybe a suitable feature to increase the compounds’ antileishmanial properties. Additionally, the existence of terminal amino groups in carbon chains also seems to be beneficial for the compound’s activity, which, due to the possibility of being easily protonated, might also correlate with the previous evidence. Finally, the hybridization of different scaffolds, once again, emerged as a promising approach to obtaining promising antileishmanial agents since, by hybridizing quinolines with other core scaffolds, one can maintain the high antileishmanial activity levels associated with the parent compounds while considerably reducing the compounds’ toxicity levels.

In conclusion, this review paper provides a considerable update on developing quinoline derivatives as promising antileishmanial agents, proving their potential for further research in this field. Furthermore, the information gathered here gives the scientific community a more in-depth understanding of the most suitable functionalizations to perform in this particular family of compounds. However, the high complexity of this parasite can still be considered one of the main limitations for developing effective antileishmanial treatments against *Leishmania*; it is almost impossible to obtain a single molecule able to not only kill the parasite but also overcome its mechanisms of defence. Finally, as mentioned before, the absence of information regarding the compounds’ mechanism of action or even the molecular target, in association with the various *Leishmania* species studied, also impairs the comparison and complete understanding of the real potential of this type of compound.

## Data Availability

Data sharing not applicable.
